# Intratumoral heterogeneity as a predictive biomarker in anti-PD-(L)1 therapies for non-small cell lung cancer

**DOI:** 10.1186/s12943-021-01331-9

**Published:** 2021-02-23

**Authors:** Wenfeng Fang, Haoxuan Jin, Huaqiang Zhou, Shaodong Hong, Yuxiang Ma, Yaxiong Zhang, Xiaofan Su, Longyun Chen, Yunpeng Yang, Shengqiang Xu, Yuwei Liao, Yuming He, Hongyun Zhao, Yan Huang, Zhibo Gao, Li Zhang

**Affiliations:** 1 Department of Medical Oncology, Sun Yat-sen University Cancer Center; State Key Laboratory of Oncology in South China; Collaborative Innovation Center for Cancer Medicine; Guangzhou Key Laboratory of Nasopharyngeal Carcinoma Diagnosis and Therapy, 651 Dongfeng Road East, Guangzhou, 510060 Guangdong China; 2 Cancer Research Institute of YuceBio, Shenzhen, China; 3 YuceBio Technology Co., Ltd., Shenzhen, China; 4 Yangjiang Key Laboratory of Respiratory Diseases, Yangjiang People’s Hospital, Yangjiang, China

## Main text

The immune checkpoint inhibitors (ICIs) have made remarkable progress in the clinical treatment of tumors in the past decade. Approximately 20% of the patients benefit from ICIs, which leads to the urgent need to identify predictive biomarkers. The acquired resistance to anti-cancer therapy is largely due to intratumoral heterogeneity (ITH). ITH is defined as an uneven distribution, spatially or temporally, of genomic diversification in an individual tumor, fostered by accumulated genetic mutations [1], and which poses a considerable challenge in the implementation of precision oncology.

ITH has been associated with the poor prognosis in solid tumors [2–5]. As known, there is no comprehensive research using ITH as a biomarker to predict the efficacy of immunotherapy in advanced non-small cell lung cancer (NSCLC) patients, due to the lack of multi-region sequencing data. In this study, we tried to use multicenter data to assess the predictive role of ITH in ICIs-treated NSCLC through a mutation frequency-based method.

## Results and discussion

### Patients characteristics

We performed whole-exome sequencing (WES) on 69 NSCLC patients, who were treated with anti-PD-(L)1 monotherapy at Sun Yat-sen University Cancer Center (SYSUCC) (Additional file 1: Supplementary materials and methods). Baseline clinicopathologic characteristics and genomic profile are summarized in Additional file 2: Fig. S2a and Additional file 3: Table S2–4, Table S6. We found that durable clinical benefit (DCB) rate, objective response rate (ORR) and median progress-free survival (mPFS) were all significantly increased in patients with high tumor mutation burden (TMB) (top 33%, cutoff =5.4 mutations/Mb; DCB rate, 52.2% vs 17.4%, Fisher’s exact test *p* = 0.005; ORR rate, 39.1% vs 10.9%, Fisher’s exact test *p* = 0.01; mPFS, 160 vs 60.5 days, log-rank *p* < 0.001, hazard ratio (HR) =2.66 [95% confidence interval (CI), 1.50–4.71]; Additional file 2: Fig. S2b-c, Fig. S3a). The same results were observed in patients with high tumor neoantigen burden (TNB) (Additional file 2: Fig. S2d-e, Fig. S3b-c).

### Intratumoral heterogeneity alone or combined with TMB can predict the efficacy of immunotherapy in the SYSUCC NSCLC cohort

**Fig. 1:**
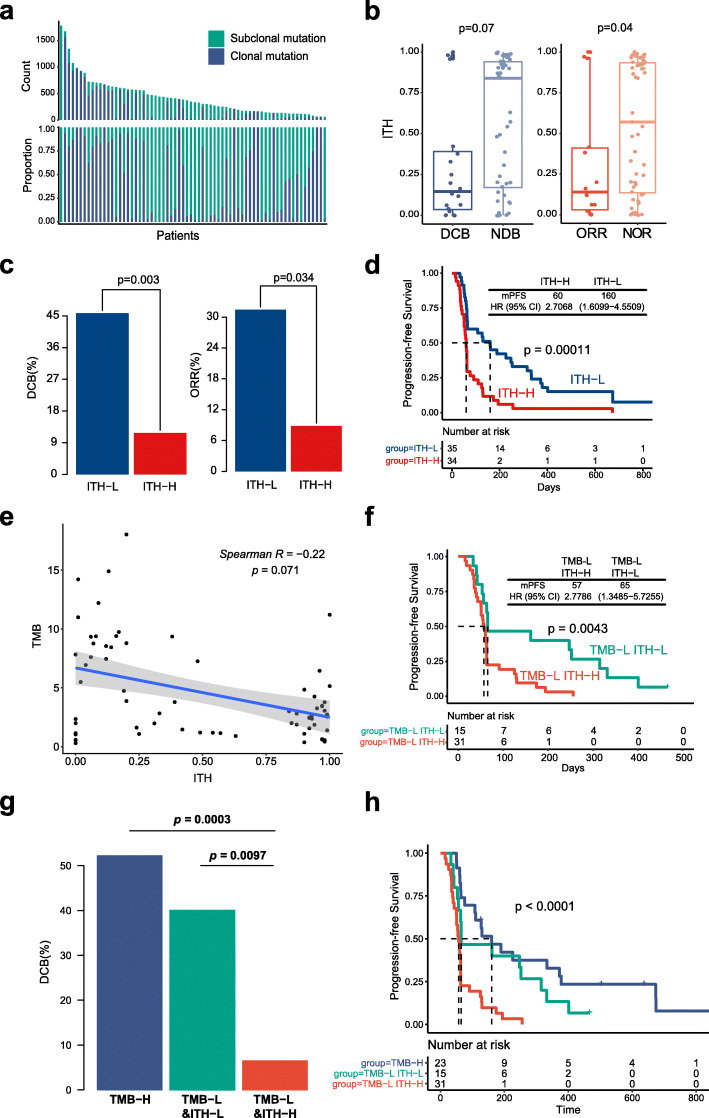
Intratumoral heterogeneity alone or combined with TMB is associated with clinical outcome in SYSUCC NSCLC cohort. **a** Distribution of intratumoral heterogeneity (ITH) in SYSUCC NSCLC cohort. Top histogram, counts of clonal mutation (indigo) and subclonal mutation (green) of each patient; lower histogram, proportion clonal mutation and subclonal mutation of each patient. **b** Boxplots of the distribution of ITH value between patients with DCB and NDB, and the distribution of ITH between patients with objective response (ORR) and non-object response (NOR). **c** Barplots of DCB rate and ORR between ITH-L group and ITH-H group. **d** ITH-L is associated with better progression-free survival. **e** The correlation between ITH and TMB. **f** ITH-L is associated with better progress-free survival in TMB-L subgroup. **g** Barplot of durable clinical benefit rate among three groups of TMB-H, TMB-L&ITH-L and TMB-L&ITH-H. **h** Progression-free survival plot among three groups of TMB-H, TMB-L&ITH-L and TMB-L&ITH-H

The ITH level for each patient was calculated and shown in Fig. 1a and Additional file 3: Table S7. DCB patients and ORR patients both had a lower ITH (*p* = 0.07 and *p* = 0.04, respectively; Fig. 1b). A higher DCB rate, ORR rate and mPFS were significant correlated with patients with lower ITH (ITH cutoff =0.45; DCB rate, 45.7% vs 11.8%, Fisher’s exact test *p* = 0.003; ORR rate, 31.4% vs 8.8%, Fisher’s exact test *p* = 0.034; mPFS, 160 vs 60 days, log-rank *p* = 0.0001, HR =2.71 [95% CI, 1.61–4.55]; Fig. 1c-d). These findings indicated that high level of ITH might be a negative predictor for ICI therapy.

There is no significant correlation between ITH and TMB in NSCLC cohort (Spearman relevance *p* = 0.07, Fig. 1e), as well as in TMB-H or TMB-L subgroup (Additional file 2: Fig. S3d-e). Most of TMB-high patients were ITH-low (*n* = 20/23, 86.96%, Additional file 2: Fig. S3f), the DCB rate, ORR rate and mPFS did not show a significant difference between the TMB-high & ITH-high group and the TMB-high & ITH-low group (Additional file 2: Fig. S4a-b). However, in the TMB-low group, a significantly higher DCB rate, ORR rate and mPFS were observed in patients with lower ITH (DCB rate, 40% vs 6.5%, Fisher’s exact test *p* = 0.0097; ORR rate, 26.7% vs 3.2%, Fisher’s exact test *p* = 0.033; mPFS, 65 vs 57 days, log-rank *p* = 0.0034, HR =2.78 [95% CI, 1.35–5.73]; Fig. 1f; Additional file 2: Fig. S4c-d). In addition, the *p*-values for interaction between TMB and ITH were 0.48 and 0.94 in all of the patients and in TMB-L group, respectively, which revealed that ITH is an independent predictor. These findings indicated that patients response to immunotherapy can be identified to the maximum extent by using the combination of ITH and TMB (Fig. 1g-h).

### Validation in the external cohorts

Our results were also validated in multiple external validation cohorts (Additional file 2: Fig. S1a, Fig. S5, Fig. S7). We found that ITH could still effectively predict the efficacy of immunotherapy in Miao’s cohort (WES) and Anagnostou’s cohort (WES), alone or combined with TMB (Additional file 2: Fig. S5a-c, Fig. S6a-c, Fig. S7a-c). Precious tumor biopsy specimens may be exhausted from routine clinical tests. In order to investigate the prediction role of ITH in addition to the WES platform, we applied the ITH measurement algorithm to circulating tumor DNA (POPLAR, OAK) data. In POPLAR/OAK cohorts, patients with low ITH showed a tendency of longer survival in POPLAR/OAK but not reach statistically significance (Additional file 2: Fig. S6d-e). Further analysis found that patients in the blood tumor mutation burden (bTMB)-H group acquired the best clinical benefit, followed by the bTMB-L & ITH-L group and bTMB-L & ITH-H group, which was consistent with the main findings in our research (Additional file 2: Fig. S5d-e, Fig. S7d-e). For comparison, we also investigate the role of ITH in the chemotherapy arm of POPLAR and OAK cohorts. The insignificant results in the chemotherapy arm suggested that ITH is a predictor for ICI therapy (Additional file 2: Fig. S9). This finding not only further validated the robustness of ITH in predicting clinical benefit of immunotherapy in NSCLC, but also benefit the future promotion and application of the ITH index with economical, versatile, and noninvasive methods.

### ITH and immunotherapy across multiple cancer types

Based on the results of the TCGA database, the distribution of ITH was significantly different in different tumors (Additional file 2: Fig. S10a). Then, we observed a tendency that the lower the median ITH level in one cancer type, the higher the ORR for its anti–PD-(L)1 therapy (Additional file 2: Fig. S10b). During the verification work using Miao’s dataset, we unexpectedly found that the predictive performance of ITH for immunotherapy also existed in this pan-cancer cohort, especially in melanoma and bladder cancer (Additional file 2: Fig. S5a, Fig. S6a, Fig. S8). We further expanded the validation cohort and found that the OS of patients with low ITH has an increasing trend in MSKCC’s pan-cancer cohort (Additional file 2: Fig. S5f, Fig. S11). The role of ITH in melanoma, esophageal and gastric cancer, head and neck cancer, and kidney cancer is acceptable, which will further expand the cancer types for potential applications of ITH.

Also, we found that in some cancer types (melanoma, NPC and renal carcinoma), TMB did not predict the benefit of ICIs, but the ITH did (Additional file 2: Fig. S5g-i). And this made us realized that ITH can be applied in substitution of TMB as a biomarker of immunotherapy in some cases when TMB fails to predict the response of immunotherapy.

### ITH and neoantigen

**Fig. 2:**
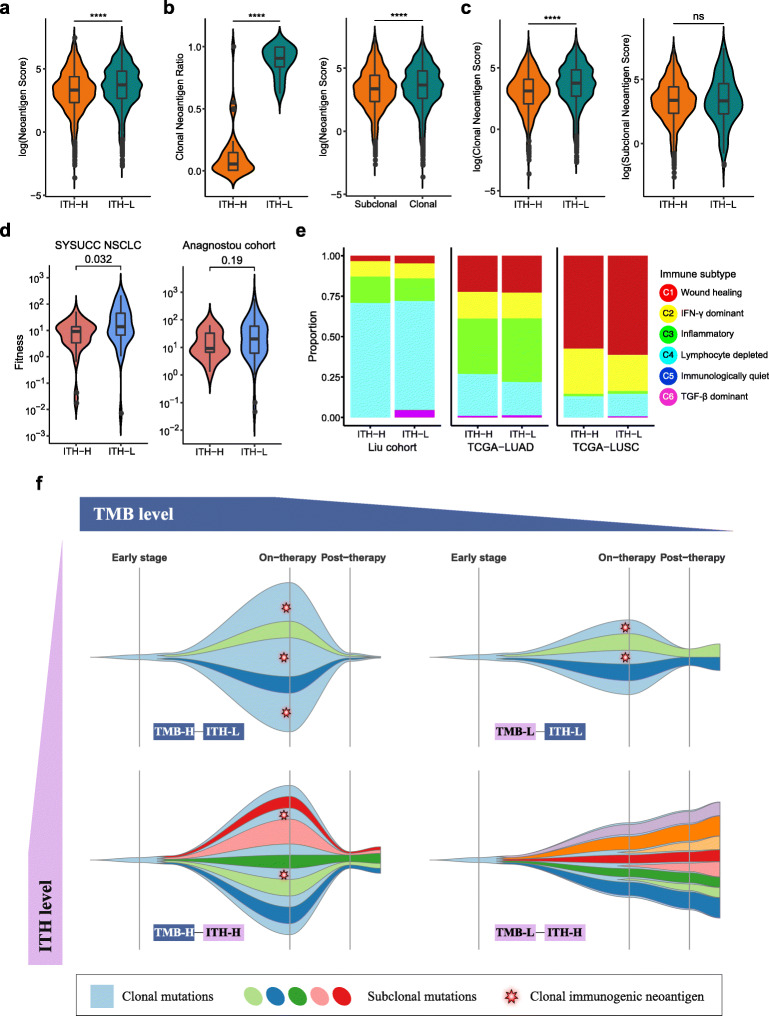
Mechanism of intratumoral heterogeneity affecting clinical outcome of immunotherapy in tumor neoantigen and microenvironment. **a** Neoantigen score of top 100 putative neoantigen of each patient between ITH-L group and ITH-H group. **b** Association between clonal neoantigen and ITH. The proportion of clonal neoantigen in patients of different ITH level is shown at left violin plot and neoantigen score difference between clonal and subclonal neoantigen is shown at right violin plot. **c** Neoantigen scores of clonal neoantigens differ across ITH groups (left) while neoantigen scores of subclonal neoantigens don’t (right). **d** Distribution of Neoantigen Fitness of each patient in ITH-H group and ITH-L group. **e** Immune subtypes proportion of ITH-H and ITH-L group in Liu cohort (left), TCGA-LUAD (middle), TCGA-LUSC (right). **f** Schematic representation of potential interplay of tumor mutation burden and intratumoral heterogeneity. High TMB is a positive factor to the response of immunotherapy while high ITH is a negative factor to the response of immunotherapy. In the situation when patient had high TMB with low ITH, the major clone in the tumor contained a relatively higher number and higher proportion of immunogenic neoantigens that responded to T cells (Top left). When patient had high TMB but with high ITH, the proportion of clonal immunogenic neoantigens decreased and patient responded less to T cells during ICIs therapy (Bottom left). When patient had low TMB with low ITH, the total number of immunogenic neoantigens decreased though the proportion of clonal immunogenic neoantigens still remained relative higher. Therefore, the clinical outcomes of TMB-L&ITH-L patients might be worse than that of TMB-H&ITH-L (Top right). However, when patient with low TMB and high ITH received the worst clinical outcome due to the small number and low proportion of clonal immunogenic neoantigens (Bottom right)

Neoantigen score was introduced to evaluate the quality of neoantigen based on mutant amino acids type, mutant peptide structure, HLA type and mutation frequency. We also introduced the Neoantigen Fitness Model to explore the capabilities of neoantigen presentation by the major histocompatibility complex (MHC) and subsequent recognition by T cells. In our NSCLC cohort, ITH-L patients had a higher proportion of clonal neoantigens and a higher score on neoantigen and MHC affinity ability. Patients with low ITH performed better in presentation and recognition of neoantigens during immunotherapy, which accounted for the better response to ICIs to some extent (Additional file 3: Table S8; Fig. 2a-d). These results were similar to previous reports [6–8]. We also performed some exploratory work on the tumor microenvironment (immune infiltrate level, cytolytic activity, immune subtype), but not found significant results (Additional file 2: Fig. S12–16; Fig. 2e).

### Limitations

The determination of the ITH threshold remains to be solved. This is likely to be related to cancer types, and to the filtering standards for mutation detection. Secondly, the study did not explore the relationship between ITH and common immunotherapy-related biomarkers, such as PD-L1 expression and microsatellite instability. Whether there is consistency between tissue and plasma based ITH estimation is also not covered in our study. Moreover, the biological explanation of how ITH function in tumor progression remain unclear. Finally, all data in this study are retrospective, and follow-up data validation is still required.

## Conclusion

In conclusion, the results of our study indicated ITH is a potential biomarker that can predict the efficacy of patients with advanced NSCLC, and even other tumors treated with ICIs. It is most prominent in low TMB populations.

## Supplementary Information

**Additional file 1:** Supplementary materials and methods

**Additional file 2: Figure S1.** Study diagram and Intratumoral heterogeneity concept. **Figure S2.** Mutation landscape and the association between tumor mutation/ neoantigen burden and clinical outcome in SYSUCC NSCLC cohort. **Figure S3.** Analysis of the biomarkers in SYSUCC NSCLC cohort. **Figure S4.** Association between TMB/ITH and clinical outcomes in SYSUCC NSCLC cohort. **Figure S5.** Validation of intratumoral heterogeneity and tumor mutation burden in predicting clinical outcome of immunotherapy in multiple cohorts. **Figure S6.** Validation of intratumoral heterogeneity in predicting clinical outcome of immunotherapy in multiple cohorts. **Figure S7.** Durable clinical benefit rate and overall survival of combination of ITH and TMB in validation cohorts. **Figure S8.** Intratumoral heterogeneity in predicting clinical outcome of immunotherapy in multiple cancer types (Miao cohort). **Figure S9.** Intratumoral heterogeneity in predicting clinical outcome of chemotherapy in POPLAR/OAK cohort. **Figure S10.** Association between intratumoral heterogeneity and objective response rate with immunotherapy across multiple cancer types. **Figure S11.** Overall survival analysis of ITH in TMB-L subgroup in MSKCC cohort. **Figure S12.** Tumor infiltrating lymphocytes analysis of different ITH groups. **Figure S13.** Difference of infiltration of each lymphocyte type in Liu cohort. **Figure S14.** Difference of infiltration of each lymphocyte type in TCGA-LUAD. **Figure S15.** Difference of infiltration of each lymphocyte type in TCGA-LUSC. **Figure S16.** Relationship between Cytolytic Activity score and ITH.

**Additional file 3: Table S1.** Detailed information of the introduced immunotherapy cohorts. **Table S2.** Summary of clinical and tumor sample characteristics. **Table S3.** Summary of Next-Generation Sequencing Analyses. **Table S4.** Somatic Sequence Alterations. **Table S5**. Summary of intratumoral heterogeneity in patients and tumor regions. **Table S6.** Copy Number Alterations. **Table S7.** Summary of calculated intratumoral heterogeneity. **Table S8.** Neoantigen Predictions

**Additional file 4: Table 1.** CCF estimation results of Miao cohort. **Table 2.** CCF estimation results of POPLAR and OAK cohorts. **Table 3.** CCF estimation results of MSKCC cohort. **Table 4.** CCF estimation results of NPC cohort. **Table 5.** CCF estimation results of ccRCC cohort.

## Data Availability

TCGA data used in the article are available from https://www.cbioportal.org/ with cancer types under PanCancer Altas subtype. MSKCC data are available from https://www.cbioportal.org/study/summary?id=msk_impact_2017. The datasets supporting the conclusions of this article are included within the article and its Additional files 3 and 4. Other relevant data are available from the corresponding authors upon reasonable request. All custom code used in this work is available from the corresponding authors upon reasonable request.

## Abbreviations

ICIs: immune checkpoint inhibitors
PD-L1: programmed death-ligand 1
TMB: tumor mutation burden
ITH: Intratumoral heterogeneity
NSCLC: non-small cell lung cancer
SYSUCC: Sun Yat-sen University Cancer Center
WES: whole-exome sequencing
PFS: Progression-free survival
ORR: Objective response rate
PR: partial response
DCB: Durable clinical benefit
TNB: Tumor neoantigen burden
mPFS: median PFS
bTMB: blood tumor mutation burden
ccRCC: clear cell renal cell carcinoma
LUAD: lung adenocarcinoma
LUSC: lung squamous cell carcinoma
NPC: nasopharyngeal carcinoma
