# Supplementary material for: Intratumoral heterogeneity as a predictive biomarker in anti-PD-(L)1 therapies for non-small cell lung cancer
Source: Mol Cancer. 2021 Feb 23;20:37. doi: 10.1186/s12943-021-01331-9 (PMC7901210; doi:10.1186/s12943-021-01331-9)
Supplement: Supplementary file 1 — Additional file 1: Supplementary materials and methods [file 12943_2021_1331_MOESM1_ESM.docx]

**Supplementary materials and methods**

**Patients and response evaluation**

The study diagram was shown in **Additional file 2: Fig. S1a**. Sixty-nine NSCLC patients were treated with anti-PD-(L)1 monotherapy at Sun Yat-sen University Cancer Center (SYSUCC)^1^. All patients enrolled in clinical trials. Eligible patients for this study were determined based on the following criteria: (i) more than 18 years old; (ii) Eastern Cooperative Oncology Group performance status: 0-1; (iii) advanced or recurrent NSCLC; (iv) failure after at least one previous line of treatment; (v) radiologically evaluable according to Response Evaluation Criteria in Solid Tumors (RECIST) version 1.1. We performed whole-exome sequencing (WES) on these patients. Computed tomography (CT) or magnetic resonance imaging (MRI) scans were reviewed. The other eight public cohorts were used, including Miao’s (WES), Anagnostou’s (WES), POPLAR/OAK (Blood-based panel), Liu’s (WES), SYSUCC nasopharyngeal carcinoma (NPC) (WES), Braun (WES), MSKCC (MSK-IMPACT panel) and TCGA (WES) cohorts^2-10^. Detailed information for these cohorts was shown in **Additional file 3: Table S1**.

Progression-free survival (PFS) was defined as the beginning of treatment to the date of disease progression (PD). Patients who had not progressed were censored at the date of their last scan. Objective response rate (ORR) was defined as the percentage of patients with complete response (CR) or partial response (PR). Durable clinical benefit (DCB) was defined as the percentage of patients who achieved CR or PR or stable disease (SD) lasting > 6 months; non-durable clinical benefit (NDB) was defined as PD or SD that lasted ≤ 6 months. The study was conducted following the declaration of Helsinki and was approved by the Ethical Review Board of SYSUCC. Informed written consent was obtained from either the subject or their guardian.

**Whole-exome sequencing and data processing**

Library Preparation and Sequencing

For each patient in our NSCLC cohort, genomic DNAs from formalin-fixed paraffin-embedded (FFPE) sections or biopsy samples and the matched blood control samples were extracted with the QIAamp DNA FFPE Tissue Kit and DNeasy Blood and tissue kit (Qiagen, USA), respectively, and quantified by Qubit 3.0 using the dsDNA HS Assay Kit (ThermoFisher Scientific, USA). Library preparations were performed with KAPA Hyper Prep Kit (KAPA Biosystems, USA). The xGen Exome Research Panel and Hybridization and Wash Reagents Kit (Integrated DNA Technology, USA) was applied for target enrichment. Sequencing was executed on Illumina HiSeq4000 platform using PE150 sequencing chemistry (Illumina, USA). The average sequence depth was 140X for tumors and 64X for normal blood controls, respectively.

Data processing of exome libraries

Low-quality and N rate beyond 10% reads were filtered out using SOAPnuke (v1.5.6). The software Burrows-Wheeler Alignment tool (BWA, Version 0.7.12) within BWA-mem algorithms was employed to align the clean reads to the human reference genome based on the UCSC hg19 build with the default parameters. SAMtools (Version 1.3) was used for alignment data conversion, sorting, and indexing. SAMBLASTER (Version 0.1.22) marked the duplicates to reduce biases in downstream analyses.

Mutation calling

Somatic mutations, including single nucleotide variants (SNVs) and small insertions and deletions (indels), were identified by VarScan (v2.4.1). All variations were identified by using Samtools mpileup with default parameter firstly. The VarScan detect somatic mutation with strict parameters, for instance, setting minimum coverage equal to 20 and minimum supporting reads equal to 3. In order to remove false-positive mutations and retrieve false-negative mutations, we performed stricter selection employing an in-house variant detection software. Each mutation was then annotated by SnpEff (Version 4.3) software. TMB was determined as the number of all nonsynonymous mutations and indels per megabase of genome examined. Microsatellite instability (MSI) status was detected using MSIsensor, and the in-house tool was then used to recalculate and correct the MSI value.

Copy number analysis and tumor purity assessment

Copy number variants (CNV) were called by exome-wide profile comparisons between tumors and matched peripheral blood using CNVkit (v0.8.1). Allele-specific copy number and tumor purity of the tumor genome were assessed using ascatNgs (v3.1.0).

**Intratumoral heterogeneity analysis**

PyClone (v0.13.0) was used to infer the cancer cell fraction (CCF) of mutations in tumors. For our WES data of NSCLC, major copy number and minor copy number of each mutation were acquired from the result of ascatNgs software. Moreover, set prior to major_copy_number. The same processes were also deployed for the validation datasets. For some samples whose copy number of each SNV and tumor purity information were not accessible, those parameters were set to the default value and set prior to total_copy_number. PyClone was deployed with 10,000 iterations and a burn-in of 1000 for all samples.

In order to evaluate the ITH level for the tumor tissue, we introduced an ITH index at the level of genetic alterations, which was defined as the proportion of the number of subclonal mutations to the total number of mutations (sum of clonal mutation number and subclonal mutation number). For each mutation (*var_i_*， i=1,…,n) in any type (SNV/Indels), the cells in the tumor can be divided into three categories: normal cells (*N_i_*), tumor cells that do not carry the mutation (*T_wt,i_*), and the Mutated tumor cells (*T_mut,i_*). For each mutation, the ratio of mutation-carrying tumor cells (*T_mut,i_*) to all tumor cells (*T_mut,i_*+*T_wt,i_*) is considered to be the tumor cell fraction at this mutation site. If similar fractions of mutated tumor cells are observed at two or more mutation sites, it can be considered that these mutation events occurred at the similar period, and they were likely to occur in the same group of tumor cells. Furthermore, a larger ratio represents the specific mutation was observed in a larger fraction of tumor cells, and the earlier occurrence of this event. Mutations of the same proportion will be divided into the same cluster, given the same cluster label (*C_j_*, j=1,…,m; m is the total number of clusters), called a tumor clone. So the estimated proportion of each tumor cell clone (*CF_Cj_*) can be calculated: *CF_Cj_* =*T_mut,Cj_* /(*T_mut,Cj_* +*T_wt,Cj_*). According to the result of PyClone, the main clone of each tumor sample (*C_main_*) was determined as the cluster with the largest CCF that contained more than one mutation. If the largest CCF cluster contained only one mutation record, then the main clone should be the mutations in both the largest cluster and the second cluster. The remain mutations were defined as subclonal mutations:


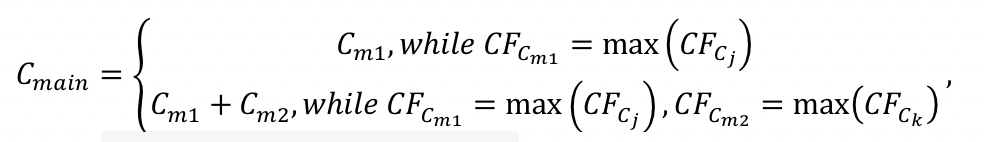


while j=1,…,m, k=1,…,m, and k≠j. The remaining clusters are determined as subclone clusters (*C_sub_*). *C_sub_*=*C_l_*, *l*∈^11^*, l*≠*j, l*≠*k.* Subsequently, the number of clonal mutations (*n_main_*) and the number of subclonal mutations (*n_sub_*) are summed in *C_main_* and *C_sub_*, respectively. The ITH index is calculated as the ratio of the number of mutations in the subclone mutation cluster to the total number of mutations: ITH= *n_sub_* / (*n_main_*+*n_sub_*)

To explore whether the ITH that measured from local sampling could be representative of the overall tumor, we collected a NSCLC cohort of 29 patients with 156 tumor regions from Zhang, et al. study and then inferred primary clone and subclones for each tumor region^12^. For overall ITH of each NSCLC patient, data from each tumor region were integrated and the frequency of mutations were recalculated, after which the tumor clonality was determined and the overall ITH index was calculated.

**Intratumoral heterogeneity measured from local sampling and the overall tumor**

To explore whether the ITH that measured from local sampling could be representative of the overall tumor, we collected a NSCLC cohort of 29 patients with 156 tumor regions from Zhang, et al. study^6^. We found that most of the ITH index of tumor regions (n=139/156, 89.1%) were distributed around the overall ITH index of the NSCLC patients, although there were a few outliers (**Additional file 2: Fig. S1b; Additional file 3: Table S5**). We found a strong correlation between the overall ITH index and the median ITH index of tumor regions of each patient (R = 0.96, p < 2.2×10-16, **Additional file 2: Fig. S1c**).

**Correlation of ITH and ORR of immune checkpoint inhibitor therapy in TCGA data**

The dataset for a total of 21 cancer types (ACC, BLCA, BRCA, CESC, COAD, READ, GBM, HNSC, KIRC, LIHC, LUAD, LUSC, MESO, OV, PAAD, PRAD, SARC, SKCM, STAD, STES, UCEC and UVM) were obtained from cBioPortal (<https://www.cbioportal.org/>). ITH was calculated for 7,087 patients in total, based on their point mutations and CNVs. The median ITH of all patients in each cancer type is considered to be the ITH value for this cancer type. The response data of ICI monotherapy for each cancer type were pooled from the largest published studies that evaluated the objective response rate and previous study^13^. For some specific cancer subtypes (i.e., *BRAF*-mut melanoma, *BRAF*-wt melanoma, triple-negative breast cancer), median ITH values are calculated after screening patients in that cancer type based on pathological or mutational information.

**Tumor neoantigen prediction**

HLA typing of the paired peripheral blood and tumor samples was performed from WES data using POLYSOLVER (v1.0) and Bwakit (v0.7.11), and the overall results were used for further neoantigen prediction. All nonsynonymous mutations and indels were translated into 21-mer peptide sequences using in-house software centered on mutated amino acid. Then, the 21-mer peptide was used to create a 9- to 11-mer peptide via a sliding window approach for prediction of MHC class I binding affinity. NetMHCpan (v3.0) was used to predict the binding strength of mutated peptides to patient-specific HLA alleles. A peptide with predicted binding affinity to any HLA allele with IC50 < 500 nM was selected. If several selected peptides were generated from the same mutation, it was only counted as one neoantigen. Tumor neoantigen burden (TNB) was determined as the number of all putative neoantigens per megabase of genome.

**Neoantigen fitness calculation**

The neoantigen fitness algorithm was applied to quantify the immune interactions of neoantigens^14^. The neoantigens that predicted by NetMHCpan (v3.0) of each patient were first aligned to reference Immune Epitope Database by Protein BLAST (blastp) software with “-outfmt 5 -evalue 100000000 -gapopen 11 -gapextend 1” parameter. Then the fitness of each neoantigen was calculated by the provided fitness pipeline^14^. Finally, the neoantigen affinity (A) and recognition (R) values of above output were used to quantify the fitness sum of each patient by manual script according to the algorithm of the neoantigen fitness model^14^.

**Immune cell infiltration inference**

The CIBERSORTx deconvolution algorithm was used to infer immune cell infiltration from RNA-seq data of Liu’s cohort, TCGA-LUAD and TCGA-LUSC, with quantile normalization disabled, and in 1,000 permutations^15^. All samples which had a P value for deconvolution >0.05 were considered to have failed deconvolution and were therefore discarded from all downstream analyses. Relative cell proportions were obtained by normalizing the CIBERSORTx output to the sample-level sum of cell counts (to obtain percentages of immune infiltration). A constant of ${10}^{-6}$ was added to all proportions in order to allow the computation of immune cell ratios. All immune cell proportions and ratios were compared using a non-parametric Wilcoxon rank-sum test for statistical significance.

**Cytolytic activity score estimation**

Cytolytic activity (CYT) score was estimated from the geometric mean of expression levels of GZMA and PRF1.

$$CYT=exp\left( \frac{\ln\left( GZMA+1 \right)+ln(PRF1+1)}{2} \right)$$

**Immune subtype identification**

Immune subtype algorithm was used to identify patients’ immune characteristic from six immune subtypes (wound healing, IFN-γ dominant, inflammatory, lymphocyte depleted, immunologically quiet and TGF-β dominant)^16^. Firstly, the expression profiles of RNA-seq from Liu’s cohort, TCGA-LUAD and TCGA-LUSC dataset were applied to calculate the possibility of each immune subtype for each patient, by using the provided tool from GitHub (<https://github.com/Gibbsdavidl/Immune-Subtype-Clustering>) with default parameter. Then the results of “BestCall” column were identified as the most likely immune subtype for each patient.

**Statistical analysis**

Categorical variables were evaluated with Fisher’s exact tests. Unpaired Mann–Whitney U test was used to compare differences for continuous variables between groups. Correlation analysis was assessed by Pearson coefficient. ROC analysis was done using the ROCR package in R. Significance of overall survival (OS) and PFS was determined via Kaplan-Meier analysis with log-rank analysis. The hazard ratio was calculated by the coxph function of the survival package in R. All statistical analysis was performed in the R statistical environment version 3.6.1. All tests were two-tailed and p-value < 0.05 was considered significant.

**References**

1. Fang W, Ma Y, Yin JC, et al. Comprehensive Genomic Profiling Identifies Novel Genetic Predictors of Response to Anti-PD-(L)1 Therapies in Non-Small Cell Lung Cancer. *Clin Cancer Res* 2019;25(16):5015-26. doi: 10.1158/1078-0432.CCR-19-0585

2. Miao D, Margolis CA, Vokes NI, et al. Genomic correlates of response to immune checkpoint blockade in microsatellite-stable solid tumors. *Nat Genet* 2018;50(9):1271-81. doi: 10.1038/s41588-018-0200-2

3. Cerami E, Gao J, Dogrusoz U, et al. The cBio cancer genomics portal: an open platform for exploring multidimensional cancer genomics data. *Cancer Discov* 2012;2(5):401-04. doi: 10.1158/2159-8290.CD-12-0095

4. Gandara DR, Paul SM, Kowanetz M, et al. Blood-based tumor mutational burden as a predictor of clinical benefit in non-small-cell lung cancer patients treated with atezolizumab. *Nat Med* 2018;24(9):1441-48. doi: 10.1038/s41591-018-0134-3

5. Gao J, Aksoy BA, Dogrusoz U, et al. Integrative analysis of complex cancer genomics and clinical profiles using the cBioPortal. *Sci Signal* 2013;6(269):l1. doi: 10.1126/scisignal.2004088

6. Zehir A, Benayed R, Shah RH, et al. Mutational landscape of metastatic cancer revealed from prospective clinical sequencing of 10,000 patients. *Nat Med* 2017;23(6):703-13. doi: 10.1038/nm.4333

7. Anagnostou V, Niknafs N, Marrone K, et al. Multimodal genomic features predict outcome of immune checkpoint blockade in non-small-cell lung cancer. *Nature Cancer* 2020;1(1):99-111. doi: 10.1038/s43018-019-0008-8

8. Fang W, Yang Y, Ma Y, et al. Camrelizumab (SHR-1210) alone or in combination with gemcitabine plus cisplatin for nasopharyngeal carcinoma: results from two single-arm, phase 1 trials. *Lancet Oncol* 2018;19(10):1338-50. doi: 10.1016/S1470-2045(18)30495-9

9. Braun DA, Hou Y, Bakouny Z, et al. Interplay of somatic alterations and immune infiltration modulates response to PD-1 blockade in advanced clear cell renal cell carcinoma. *Nature Medicine* 2020 doi: 10.1038/s41591-020-0839-y

10. Liu D, Schilling B, Liu D, et al. Integrative molecular and clinical modeling of clinical outcomes to PD1 blockade in patients with metastatic melanoma. *Nature medicine* 2019;25(12):1916-27. doi: 10.1038/s41591-019-0654-5

11. Yang X, Qi C, Ji M. PD-L1 Status and Survival in Patients With Lung Cancer. *JAMA* 2019;322(8):783. doi: 10.1001/jama.2019.9177

12. Zhang Y, Chang L, Yang Y, et al. Intratumor heterogeneity comparison among different subtypes of non-small-cell lung cancer through multi-region tissue and matched ctDNA sequencing. *Mol Cancer* 2019;18(1):7. doi: 10.1186/s12943-019-0939-9 [published Online First: 2019/01/11]

13. Yarchoan M, Hopkins A, Jaffee EM. Tumor Mutational Burden and Response Rate to PD-1 Inhibition. *N Engl J Med* 2017;377(25):2500-01. doi: 10.1056/NEJMc1713444

14. Łuksza M, Riaz N, Makarov V, et al. A neoantigen fitness model predicts tumour response to checkpoint blockade immunotherapy. *Nature* 2017;551(7681):517-20. doi: 10.1038/nature24473

15. Newman AM, Steen CB, Liu CL, et al. Determining cell type abundance and expression from bulk tissues with digital cytometry. *Nature Biotechnology* 2019;37(7):773-82. doi: 10.1038/s41587-019-0114-2

16. Thorsson V, Gibbs DL, Brown SD, et al. The Immune Landscape of Cancer. *Immunity* 2018;48(4) doi: 10.1016/j.immuni.2018.03.023
