# Supplementary material for: Intratumoral heterogeneity as a predictive biomarker in anti-PD-(L)1 therapies for non-small cell lung cancer
Source: Mol Cancer. 2021 Feb 23;20:37. doi: 10.1186/s12943-021-01331-9 (PMC7901210; doi:10.1186/s12943-021-01331-9)
Supplement: Supplementary file 2 — Additional file 2: Figure S1. Study diagram and Intratumoral heterogeneity concept. Figure S2. Mutation landscape and the association between tumor mutation/ neoantigen burden and clinical outcome in SYSUCC NSCLC cohort. Figure S3. Analysis of the biomarkers in SYSUCC NSCLC cohort. Figure S4. Association between TMB/ITH and clinical outcomes in SYSUCC NSCLC cohort. Figure S5. Validation of intratumoral heterogeneity and tumor mutation burden in predicting clinical outcome of immunotherapy in multiple cohorts. Figure S6. Validation of intratumoral heterogeneity in predicting clinical outcome of immunotherapy in multiple cohorts. Figure S7. Durable clinical benefit rate and overall survival of combination of ITH and TMB in validation cohorts. Figure S8. Intratumoral heterogeneity in predicting clinical outcome of immunotherapy in multiple cancer types (Miao cohort). Figure S9. Intratumoral heterogeneity in predicting clinical outcome of chemotherapy in POPLAR/OAK cohort. Figure S10. Association between intratumoral heterogeneity and objective response rate with immunotherapy across multiple cancer types. Figure S11. Overall survival analysis of ITH in TMB-L subgroup in MSKCC cohort. Figure S12. Tumor infiltrating lymphocytes analysis of different ITH groups. Figure S13. Difference of infiltration of each lymphocyte type in Liu cohort. Figure S14. Difference of infiltration of each lymphocyte type in TCGA-LUAD. Figure S15. Difference of infiltration of each lymphocyte type in TCGA-LUSC. Figure S16. Relationship between Cytolytic Activity score and ITH. [file 12943_2021_1331_MOESM2_ESM.pdf]

Supplementary Figures

Fig. S1.

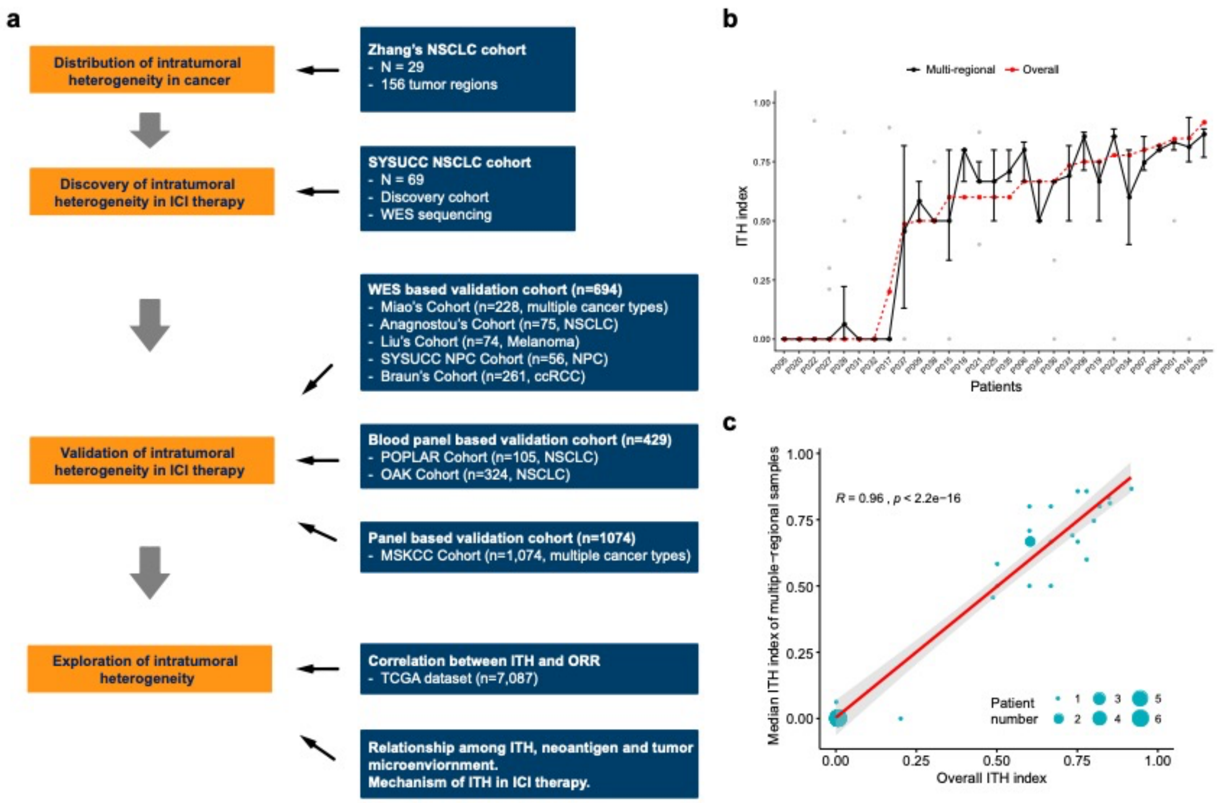

Fig. S1. Study diagram and Intratumoral heterogeneity concept. a, the study diagram. **b-c,** Intratumoral heterogeneity (ITH) measured from local sampling could be representative of the overall tumor.

Fig. S2.

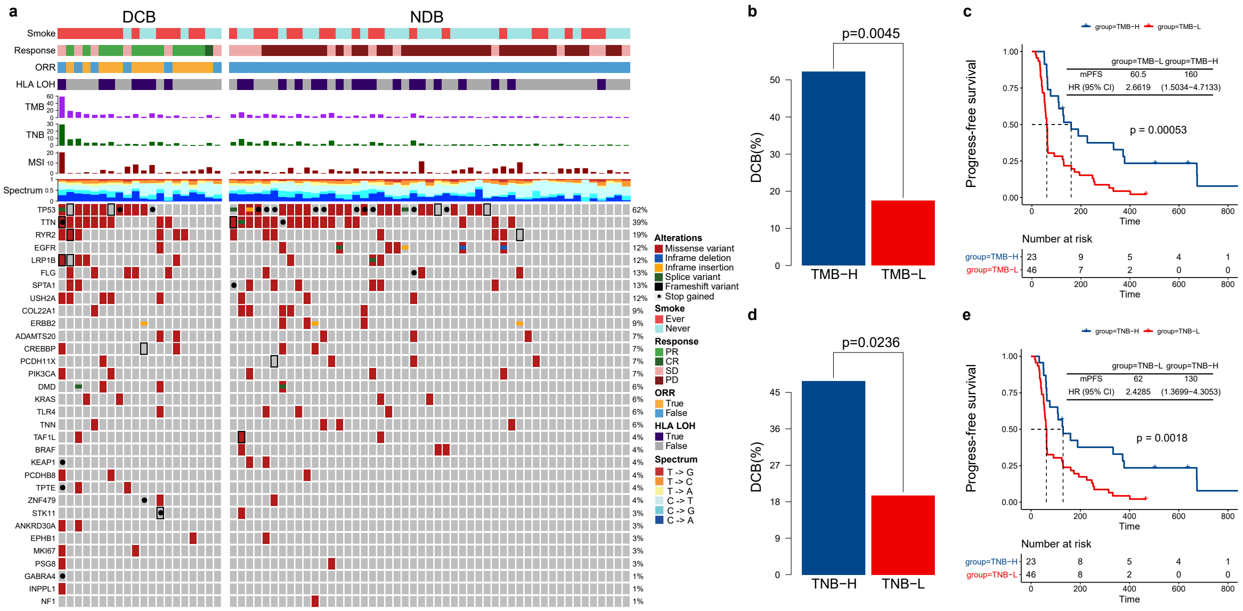

Fig. S2. Mutation landscape and the association between tumor mutation/ neoantigen burden and clinical outcome in SYSUCC NSCLC cohort. a, mutation landscape of SYSUCC NSCLC cohort. Top tracks, indication of smoking status, clinical outcome and type I HLA loss of heterogeneity (LOH) status of each patient. Top histogram, tumor mutation burden (TMB), tumor neoantigen burden (TNB) and microsatellite instability (MSI) values of each patient, and mutation spectrum of each patient. Center heatmap, distribution of non-synonymous driver mutation events; right annotation, frequency of somatic alterations. Patients were divided into durable clinical benefit (DCB) group and non-durable clinical benefit (NDB) group. **b**, barplot of durable clinical benefit rate between TMB-H group and TMB-L group. **c**, TMB-H is associated with better progression-free survival. **d**, barplot durable clinical benefit rate between TNB-H group and TNB-L group. **e**, TNB-H is associated with better progression-free survival.

Fig. S3.

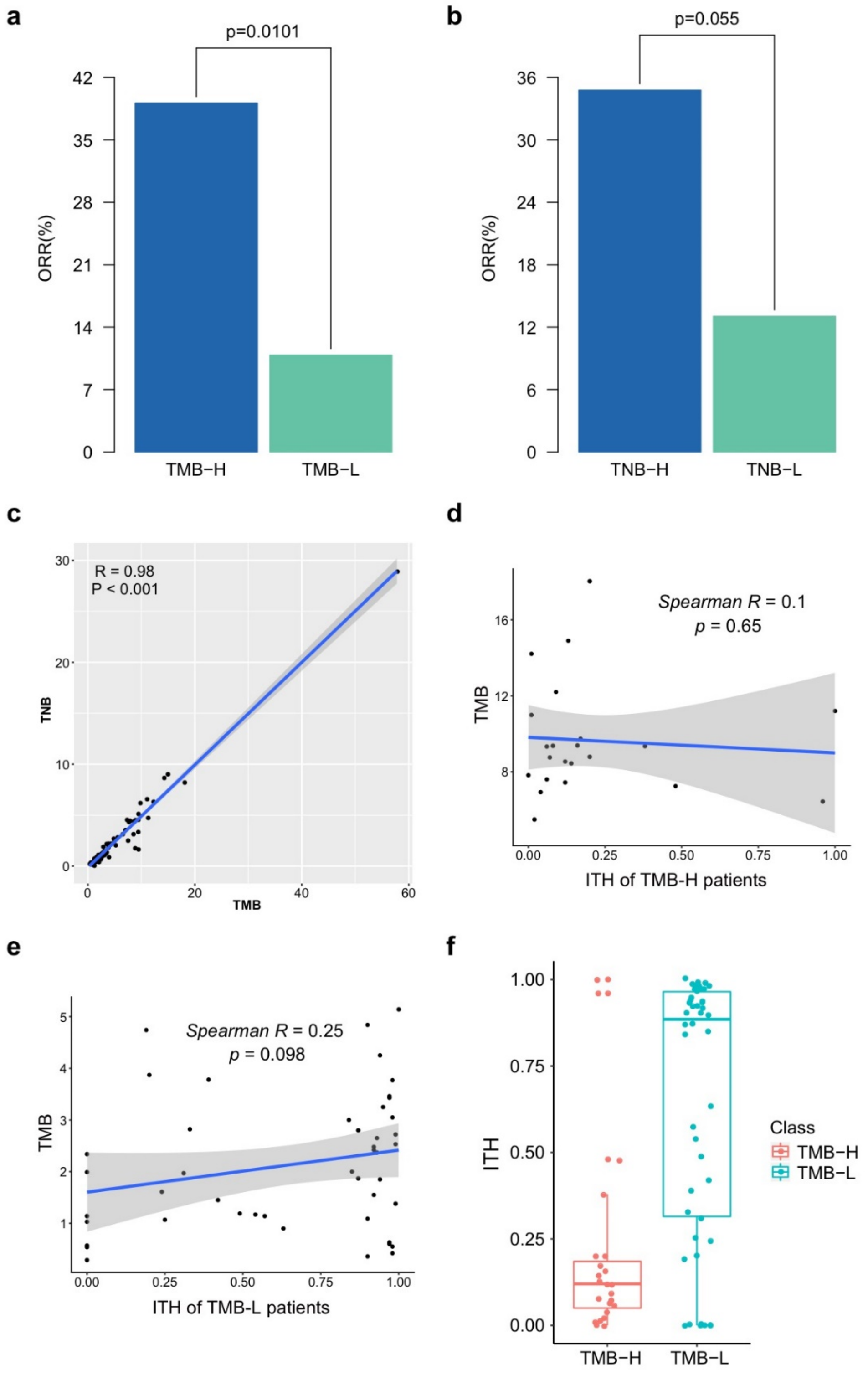

a
p=0.0101
ORR(%)
42
35
28
21
14
7
0
TMB−H
TMB−L
b
p=0.055
ORR(%)
36
30
24
18
12
6
0
TNB−H
TNB−L
c
R = 0.98
P < 0.001
TNB
TMB
d
Spearman R = 0.1
p = 0.65
TMB
ITH of TMB-H patients
e
Spearman R = 0.25
p = 0.098
TMB
ITH of TMB-L patients
f
ITH
Class
TMB−H
TMB−L

Fig. S3. Analysis of the biomarkers in SYSUCC NSCLC cohort. a, Barplot of objective response rate between different TMB groups. **b**, Barplot of objective response rate between different TNB groups. **c**, Correlation between TMB and TNB. **d**, Correlation between ITH and TMB in TMB-H subgroup. **e**, Correlation between ITH and TMB in TMB-L subgroup. **f**, Distribution of ITH in TMB-H group and TMB-L group.

Fig. S4.

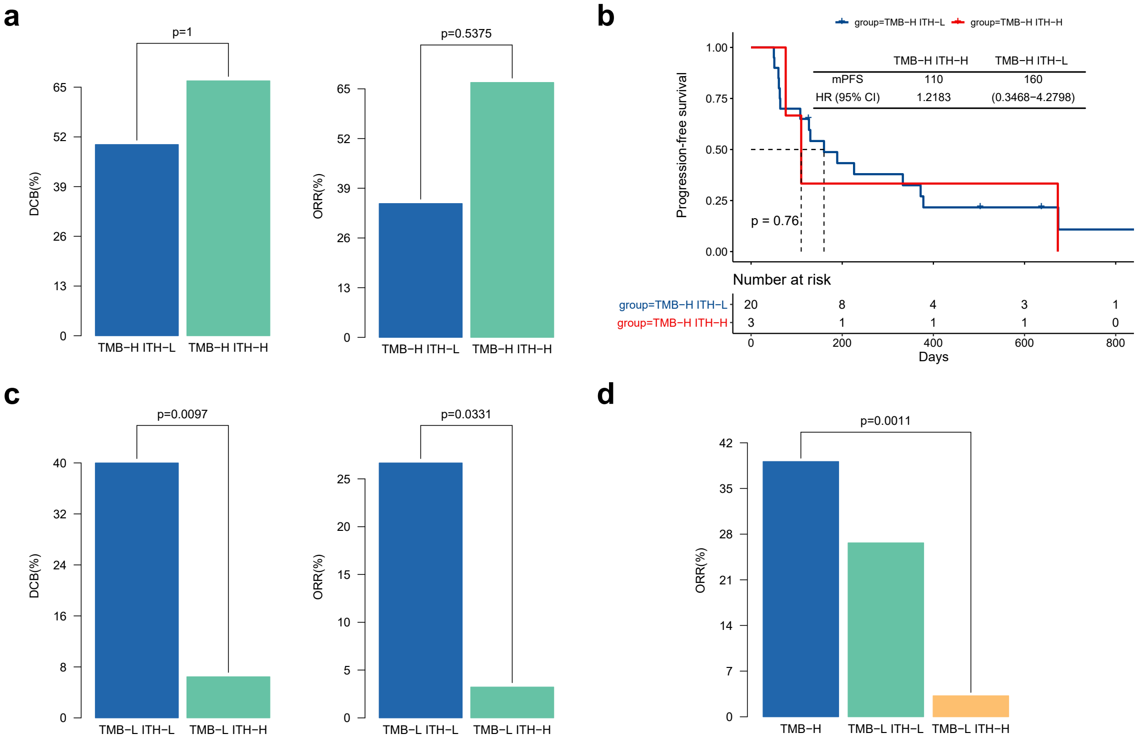

Fig. S4. Association between TMB/ITH and clinical outcomes in SYSUCC NSCLC cohort. **a**, Barplot of durable clinical benefit rate (left) and objective response rate (right) between ITH-L and ITH-H in TMB-H subgroup. **b**, Progression-free survival plot of different ITH level in TMB-H subgroup. **c**, Barplot of durable clinical benefit rate (left) and objective response rate (right) between ITH-L and ITH-H in TMB-L subgroup. **d**, Objective response rate among TMB−H, TMB−L&ITH−L and TMB−L&ITH−H three groups.

Fig. S5.

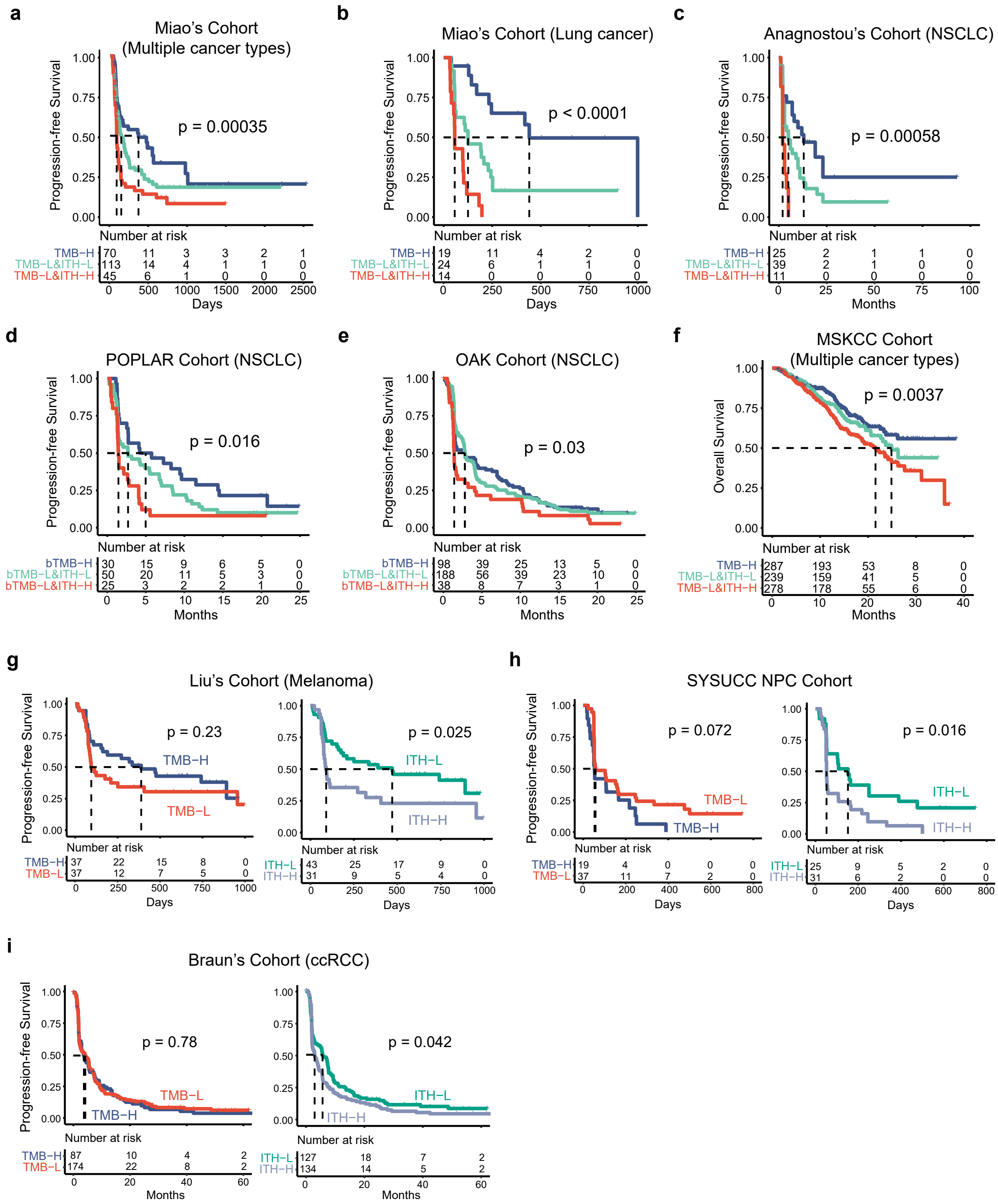
a
Miao's Cohort (Multiple cancer types)
p = 0.00035
Progression-free Survival
1.00
0.75
0.50
0.25
0.00
Number at risk
TMB−H 70 11 3 3 2 1
TMB−L&ITH−L 113 14 4 1 1 0
TMB−L&ITH−H 45 6 1 0 0 0
0 500 1000 1500 2000 2500
Days
b
Miao's Cohort (Lung cancer)
p < 0.0001
Progression-free Survival
Number at risk
TMB−H 19 11 4 2 0
TMB−L&ITH−L 24 6 1 1 0
TMB−L&ITH−H 14 0 0 0 0
0 250 500 750 1000
Days
c
Anagnostou's Cohort (NSCLC)
p = 0.00058
Progression-free Survival
Number at risk
TMB−H 25 2 1 1 0
TMB−L&ITH−L 39 2 1 0 0
TMB−L&ITH−H 11 0 0 0 0
0 25 50 75 100
Months
d
POPLAR Cohort (NSCLC)
p = 0.016
Progression-free Survival
Number at risk
bTMB−H 30 15 9 6 5 0
bTMB−L&ITH−L 50 20 11 5 3 0
bTMB−L&ITH−H 25 3 2 2 1 0
0 5 10 15 20 25
Months
e
OAK Cohort (NSCLC)
p = 0.03
Progression-free Survival
Number at risk
bTMB−H 98 39 25 13 5 0
bTMB−L&ITH−L 188 56 39 23 10 0
bTMB−L&ITH−H 38 8 7 3 1 0
0 5 10 15 20 25
Months
f
MSKCC Cohort (Multiple cancer types)
p = 0.0037
Overall Survival
Number at risk
TMB−H 287 193 53 8 0
TMB−L&ITH−L 239 159 41 5 0
TMB−L&ITH−H 278 178 55 6 0
0 10 20 30 40
Months
g
Liu's Cohort (Melanoma)
p = 0.23
TMB−H
TMB−L
Progression-free Survival
Number at risk
TMB−H 37 22 15 8 0
TMB−L 37 12 7 5 0
0 250 500 750 1000
Days
p = 0.025
ITH−L
ITH−H
Number at risk
ITH−L 43 25 17 9 0
ITH−H 31 9 5 4 0
0 250 500 750 1000
Days
h
SYSUCC NPC Cohort
p = 0.072
TMB−L
TMB−H
Progression-free Survival
Number at risk
TMB−H 19 4 0 0 0
TMB−L 37 11 7 2 0
0 200 400 600 800
Days
p = 0.016
ITH−L
ITH−H
Number at risk
ITH−L 25 9 5 2 0
ITH−H 31 6 2 0 0
0 200 400 600 800
Days
i
Braun's Cohort (ccRCC)
p = 0.78
TMB−L
TMB−H
Progression-free Survival
Number at risk
TMB−H 87 10 4 2
TMB−L 174 22 8 2
0 20 40 60
Months
p = 0.042
ITH−L
ITH−H
Number at risk
ITH−L 127 18 7 2
ITH−H 134 14 5 2
0 20 40 60
Months

Fig. S5. Validation of intratumoral heterogeneity and tumor mutation burden in predicting clinical outcome of immunotherapy in multiple cohorts. a-e, progression-free survival plot among three groups of TMB-H, TMB-L&ITH-L and TMB-L&ITH-H in Miao cohort (**a**), Miao lung cancer cohort (**b**), Anagnostou cohort (**c**), POPLAR cohort (**d**) and OAK cohort (**e**). **f**, overall survival plot among three groups of TMB-H, TMB-L&ITH-L and TMB-L&ITH-H in MSKCC cohort. **g-i**, TMB is not associated with clinical outcome but ITH still can predict the efficacy of immunotherapy in Liu cohort (**g**), SYSUCC NPC cohort (**h**) and Braun cohort (**i**).

Fig. S6.

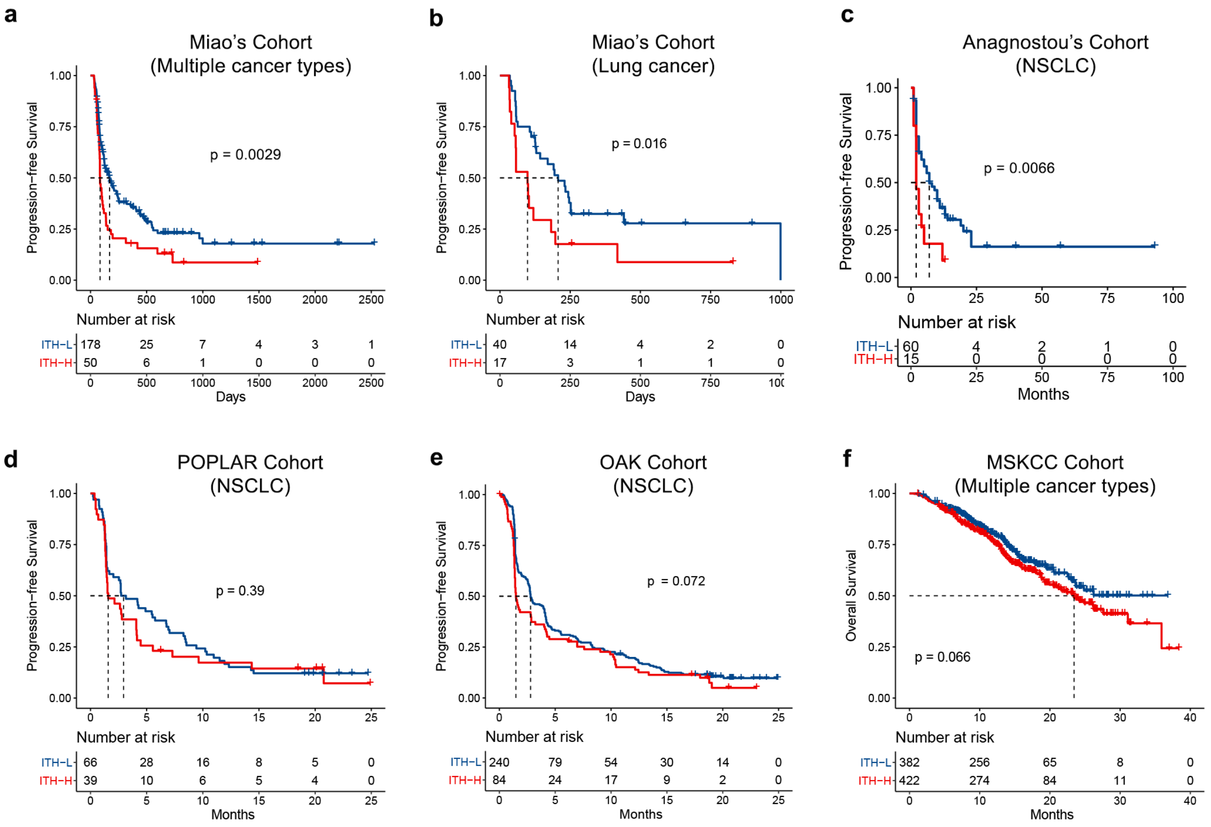

Fig. S6. Validation of intratumoral heterogeneity in predicting clinical outcome of immunotherapy in multiple cohorts. a-e, progression-free survival plot among three groups of ITH-L and ITH-H in Miao cohort (**a**), Miao lung cancer cohort (**b**), Anagnostou cohort (**c**), POPLAR cohort (**d**) and OAK cohort (**e**). **f**, overall survival plot among ITH-L and ITH-H in MSKCC cohort.

Fig. S7.

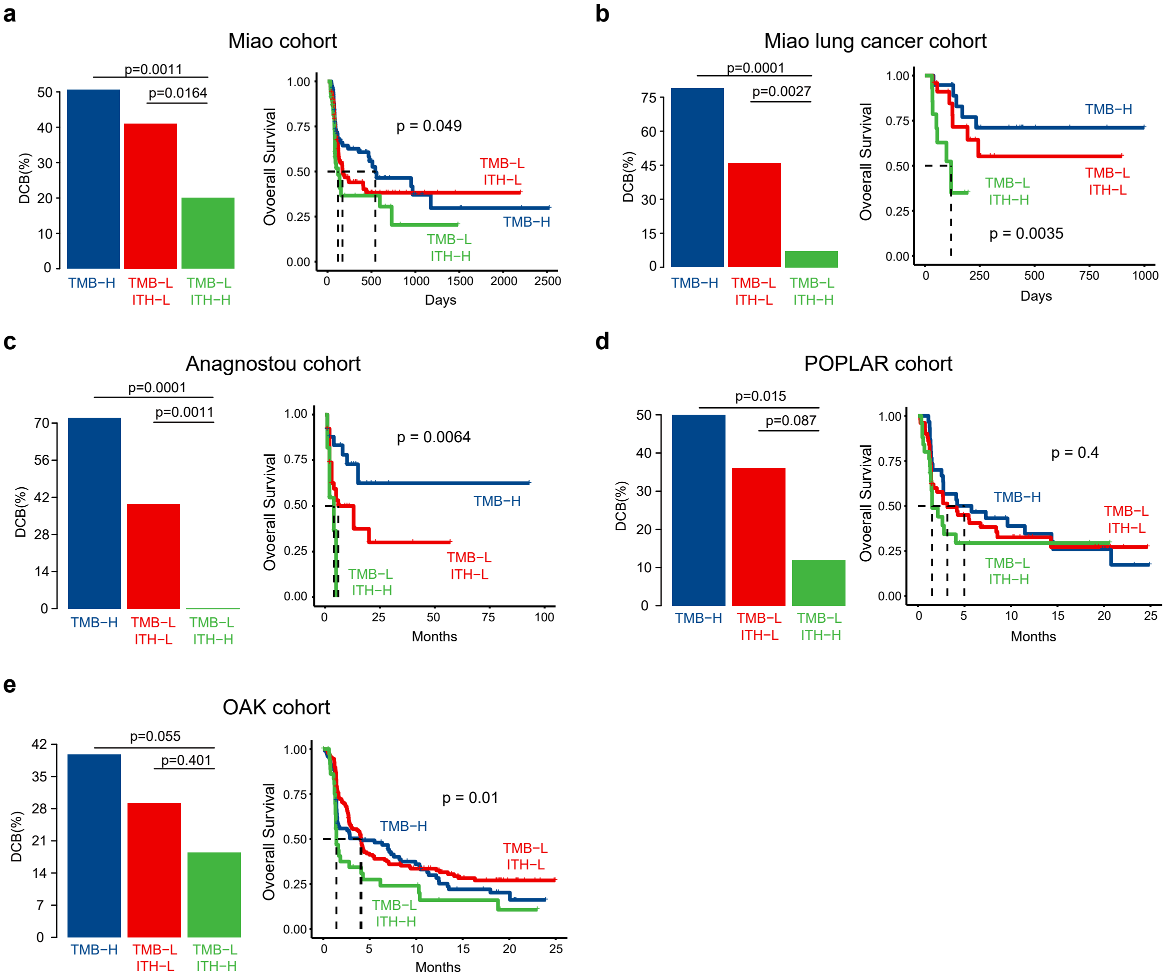

Fig. S7. Durable clinical benefit rate and overall survival of combination of ITH and TMB in validation cohorts. a-e, Barplot of durable clinical benefit rate and overall survival among TMB−H, TMB−L&ITH−L and TMB−L&ITH−H three groups in Miao cohort (**a**), Miao lung cancer cohort (**b**), Anagnostou cohort (**c**), POPLAR cohort (**d**) and OAK cohort (**e**).

Fig. S8.

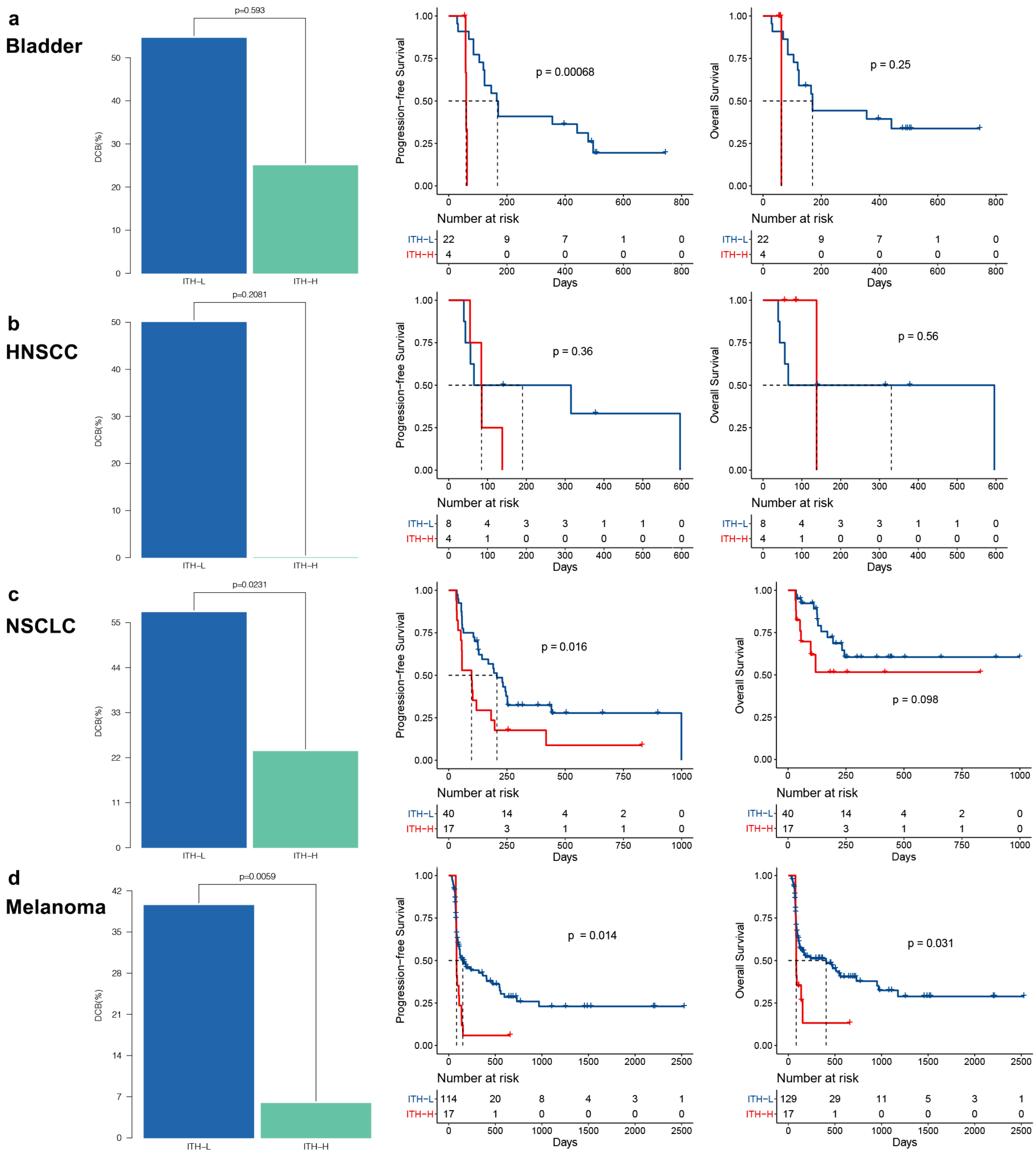

Fig. S8. Intratumoral heterogeneity in predicting clinical outcome of immunotherapy in multiple cancer types (Miao cohort). a-d, Bladder cancer (**a**), HNSCC (**b**), NSCLC (**c**), Melanoma (**d**).

Fig. S9.

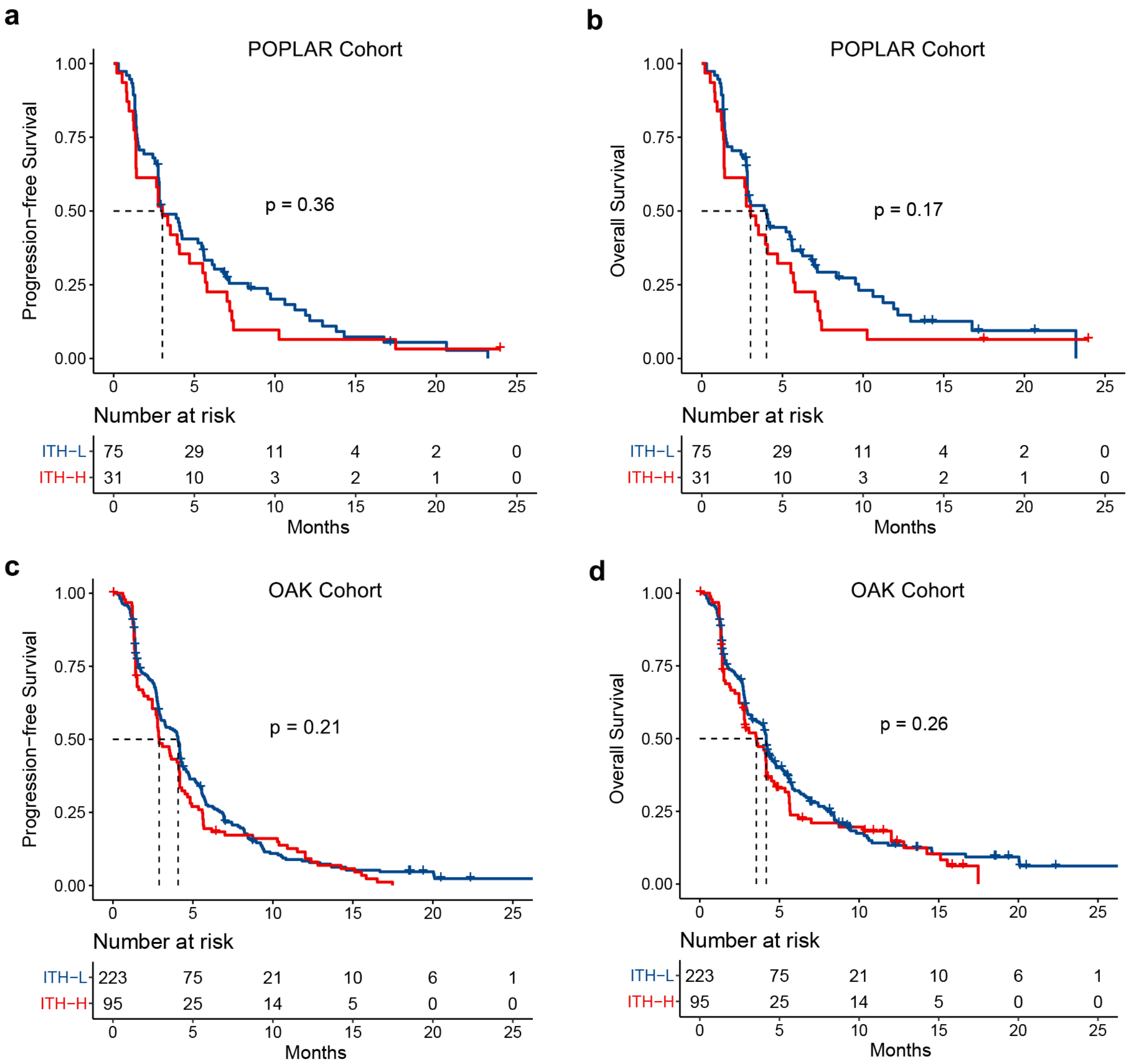

Fig. S9. Intratumoral heterogeneity in predicting clinical outcome of chemotherapy in POPLAR/OAK cohort.

Fig. S10.

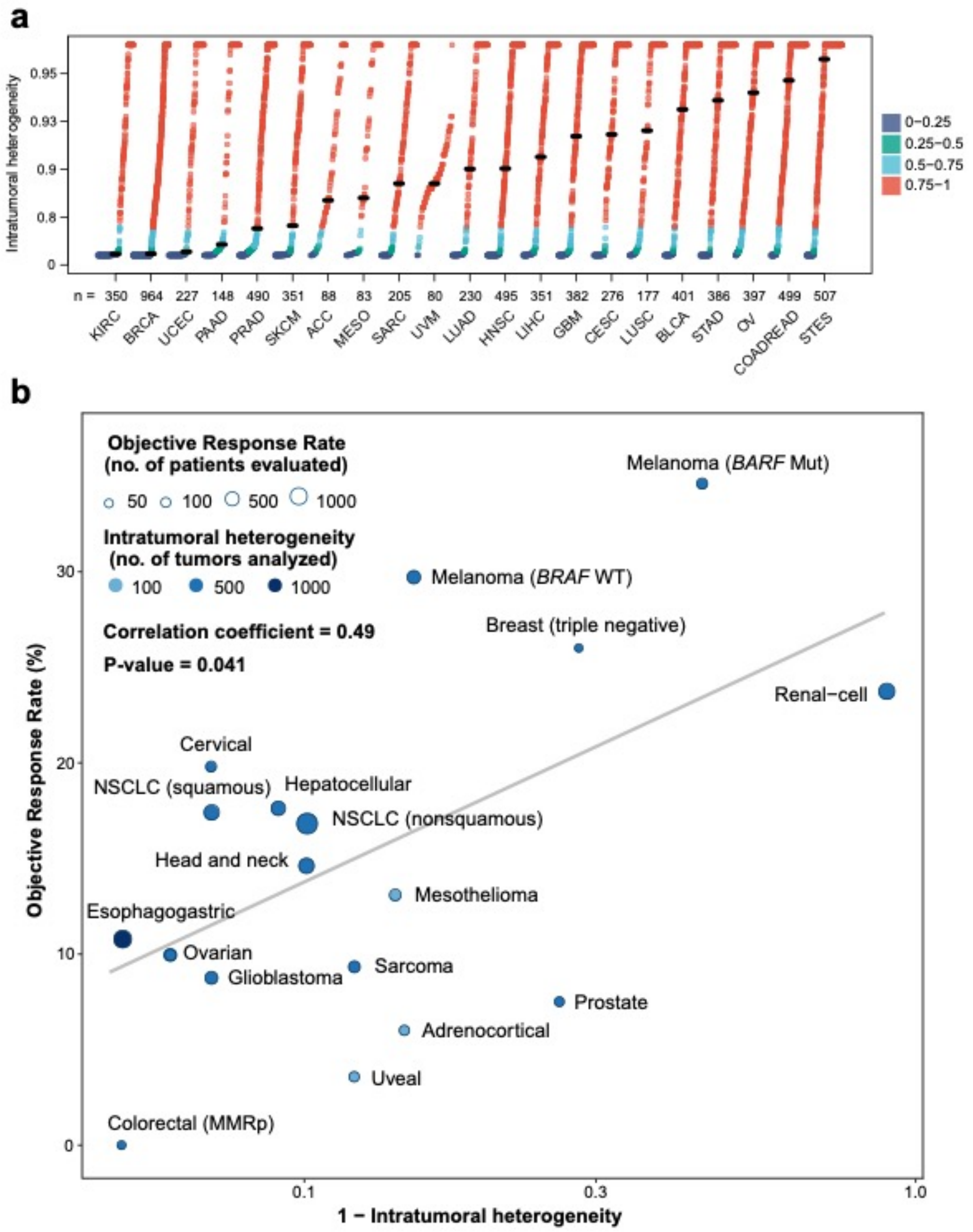
a
Intratumoral heterogeneity
0.95
0.93
0.9
0.8
0
0-0.25
0.25-0.5
0.5-0.75
0.75-1
n = 350 964 227 148 490 351 88 83 205 80 230 495 351 382 276 177 401 386 397 499 507
KIRC BRCA UCEC PAAD PRAD SKCM ACC MESO SARC UVM LUAD HNSC LIHC GBM CESC LUSC BLCA STAD OV COADREAD STES
b
Objective Response Rate (no. of patients evaluated)
50 100 500 1000
Intratumoral heterogeneity (no. of tumors analyzed)
100 500 1000
Correlation coefficient = 0.49
P-value = 0.041
Melanoma (BARF Mut)
Melanoma (BRAF WT)
Breast (triple negative)
Renal-cell
Cervical
NSCLC (squamous)
Hepatocellular
NSCLC (nonsquamous)
Head and neck
Mesothelioma
Esophagogastric
Ovarian
Glioblastoma
Sarcoma
Prostate
Adrenocortical
Uveal
Colorectal (MMRp)
Objective Response Rate (%)
30
20
10
0
0.1
0.3
1.0
1 − Intratumoral heterogeneity

Fig. S10. Association between intratumoral heterogeneity and objective response rate with immunotherapy across multiple cancer types. a, distribution of ITH among 21 cancer types from TCGA dataset. Data on the y axis are shown on a logarithmic scale. Median ITH of each cancer type is label in black and is rank in descending order. **b**, Correlation between intratumoral heterogeneity and objective response rate with immunotherapy in 17 cancer types. The number of patients who were evaluated for the objective response rate is shown for each tumor type (size of the circle), along with the number of tumor samples that were analyzed to calculate the ITH (degree of shading of the circle). Data on the x axis are shown on a logarithmic scale. MMRp denotes mismatch repair-proficient, NSCLC non–small-cell lung cancer, *BRAF* WT *BRAF* wild type, and *BARF* Mut *BRAF* mutant type.

Fig. S11.

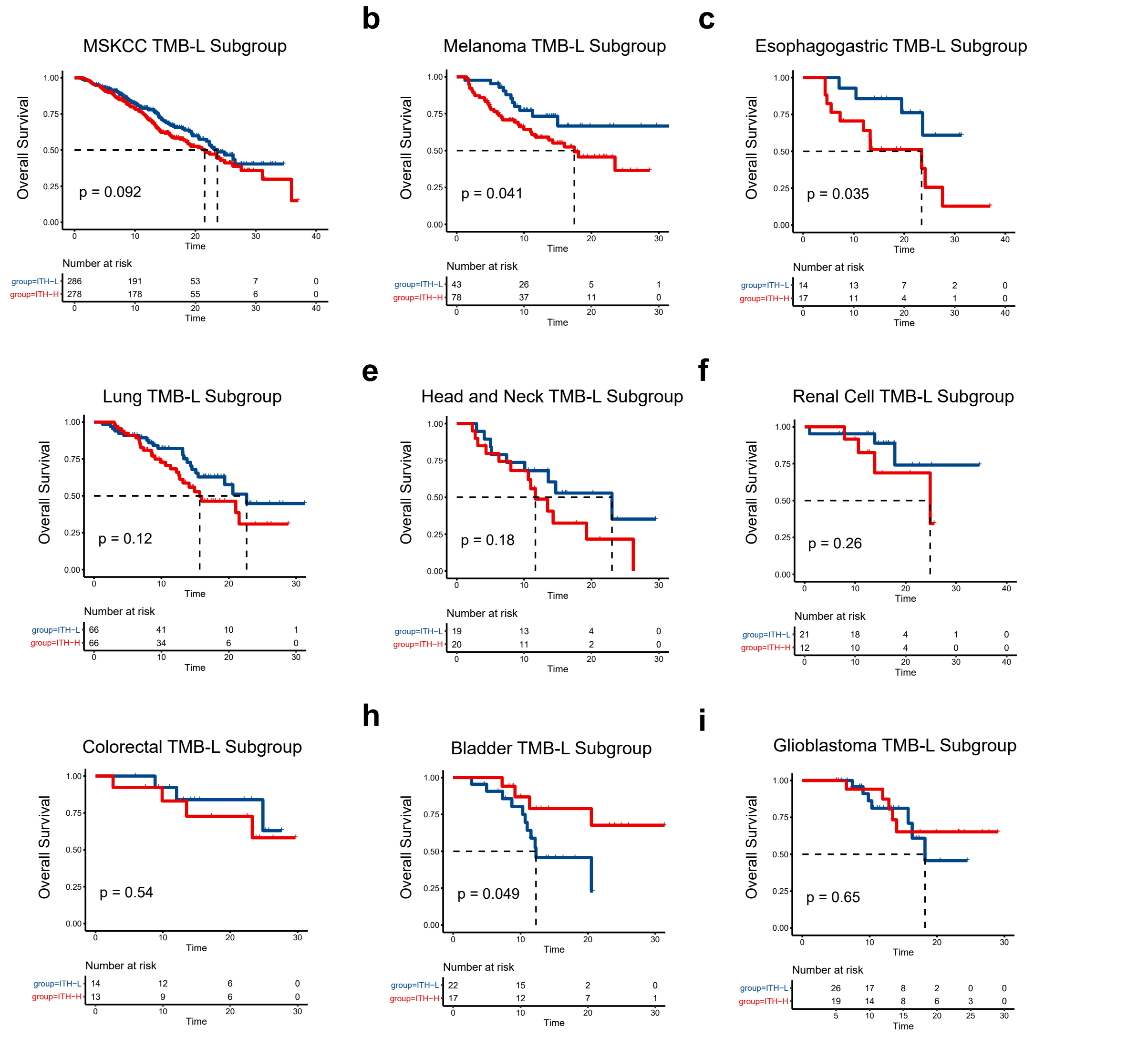

Fig. S11. Overall survival analysis of ITH in TMB-L subgroup in MSKCC cohort. a, Overall survival of ITH in all TMB-L patients. **b-i**, Overall survival of ITH in TMB-L patients in eight majority cancer types of MSKCC cohort, including Melanoma (**b**), Esophagogastric (**c**), Lung (**d**), Head and Neck (**e**), Renal Cell (**f**), Colorectal (**g**), Bladder (**h**) and Glioblastoma (**i**).

Fig. S12.

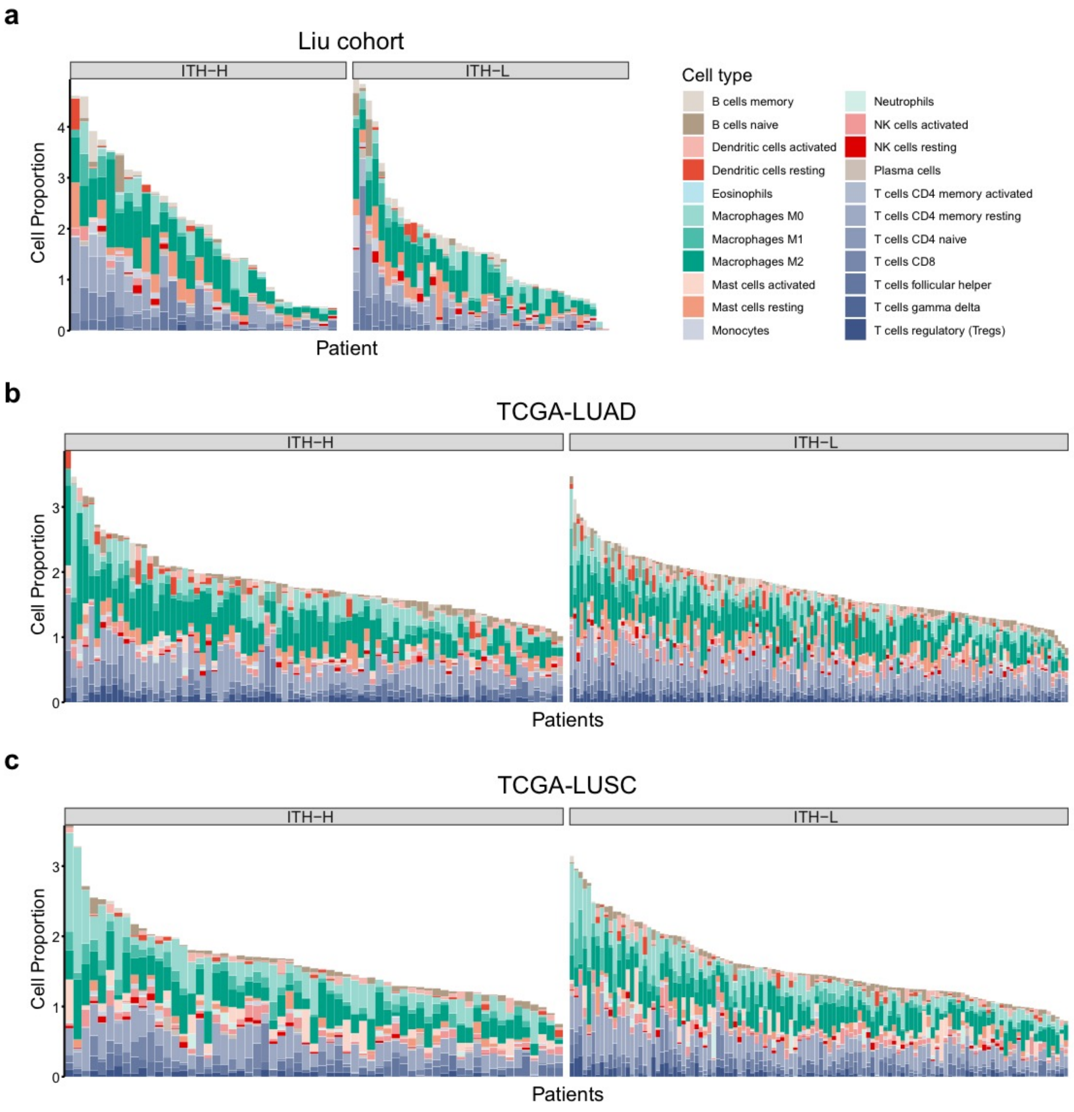

Fig. S12. Tumor infiltrating lymphocytes analysis of different ITH groups. a-c, Fraction of tumor infiltrating lymphocytes (TILs) of patients in ITH-H group and ITH-L group in Liu cohort (**a**), TCGA-LUAD (**b**) and TCGA-LUSC (**c**).

Fig. S13.

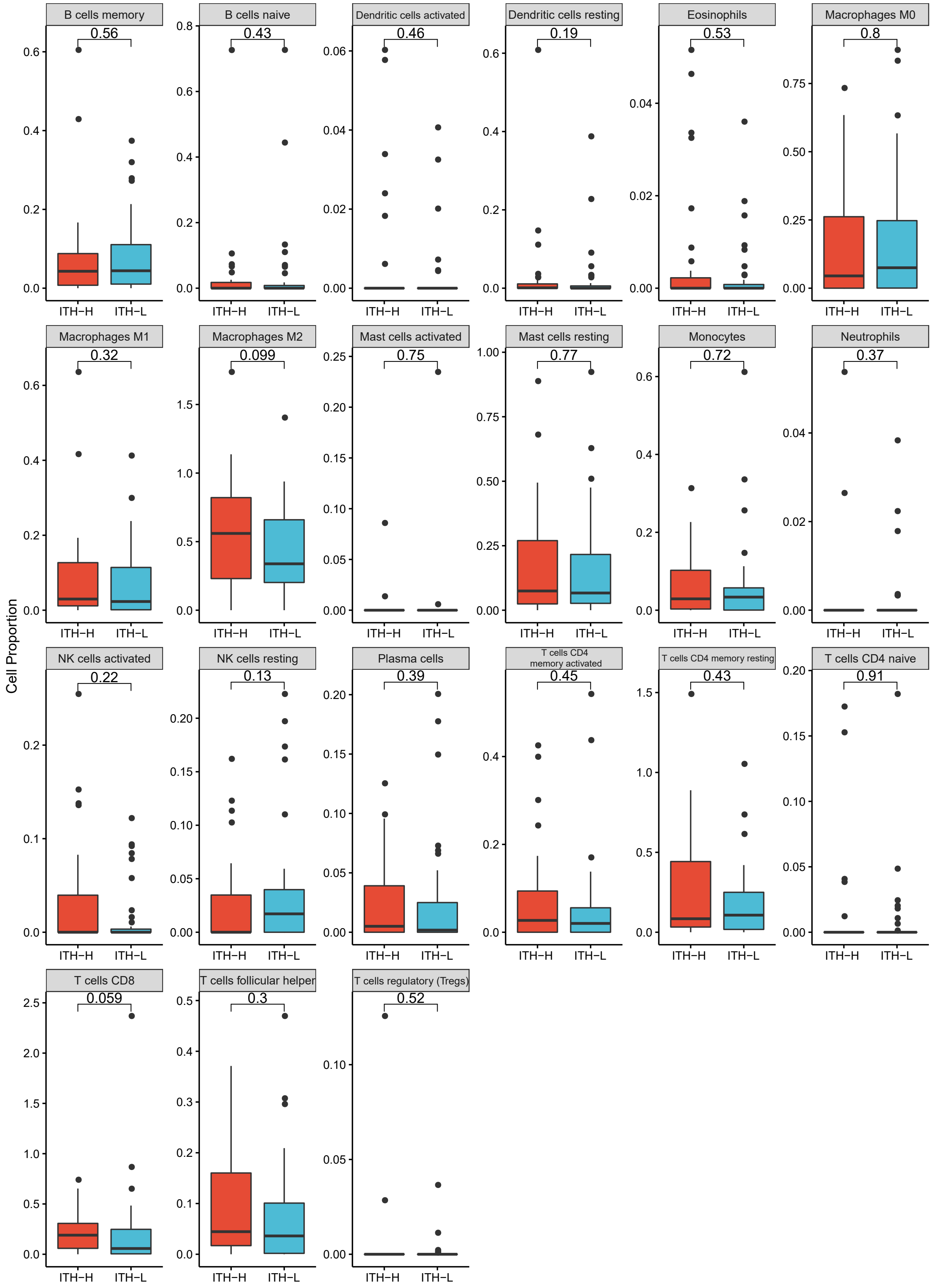
B cells memory
0.56
B cells naive
0.43
Dendritic cells activated
0.46
Dendritic cells resting
0.19
Eosinophils
0.53
Macrophages M0
0.8
Macrophages M1
0.32
Macrophages M2
0.099
Mast cells activated
0.75
Mast cells resting
0.77
Monocytes
0.72
Neutrophils
0.37
NK cells activated
0.22
NK cells resting
0.13
Plasma cells
0.39
T cells CD4 memory activated
0.45
T cells CD4 memory resting
0.43
T cells CD4 naive
0.91
T cells CD8
0.059
T cells follicular helper
0.3
T cells regulatory (Tregs)
0.52
Cell Proportion
ITH−H
ITH−L

Fig. S13. Difference of infiltration of each lymphocyte type in Liu cohort. Difference of infiltration of each lymphocyte type between ITH-H group and ITH-L group in Liu cohort.

Fig. S14.

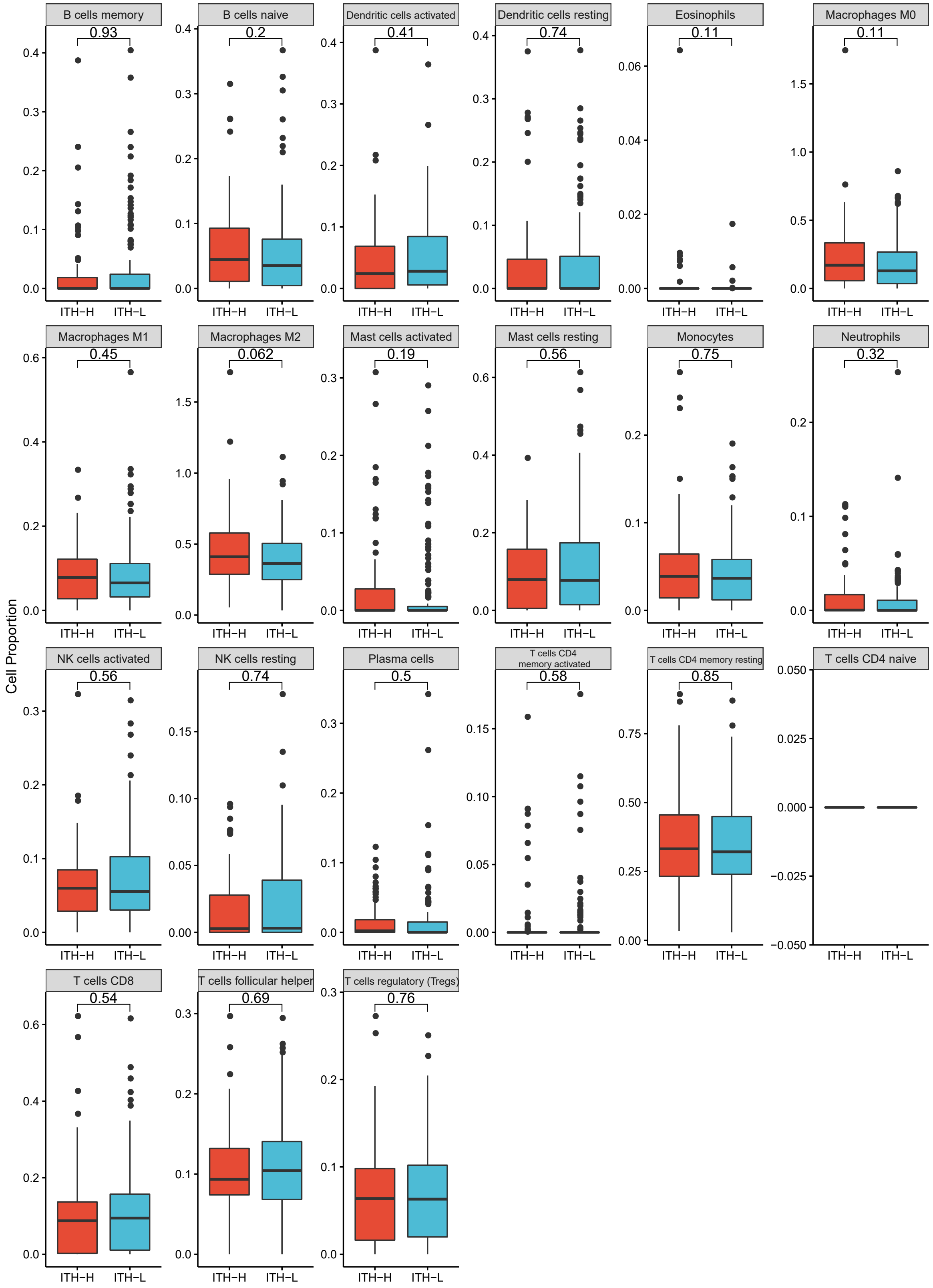

Cell Proportion
B cells memory
0.93
B cells naive
0.2
Dendritic cells activated
0.41
Dendritic cells resting
0.74
Eosinophils
0.11
Macrophages M0
0.11
Macrophages M1
0.45
Macrophages M2
0.062
Mast cells activated
0.19
Mast cells resting
0.56
Monocytes
0.75
Neutrophils
0.32
NK cells activated
0.56
NK cells resting
0.74
Plasma cells
0.5
T cells CD4 memory activated
0.58
T cells CD4 memory resting
0.85
T cells CD4 naive
T cells CD8
0.54
T cells follicular helper
0.69
T cells regulatory (Tregs)
0.76
ITH−H
ITH−L

Fig. S14. Difference of infiltration of each lymphocyte type in TCGA-LUAD. Difference of infiltration of each lymphocyte type between ITH-H group and ITH-L group in TCGA-LUAD.

Fig. S15.

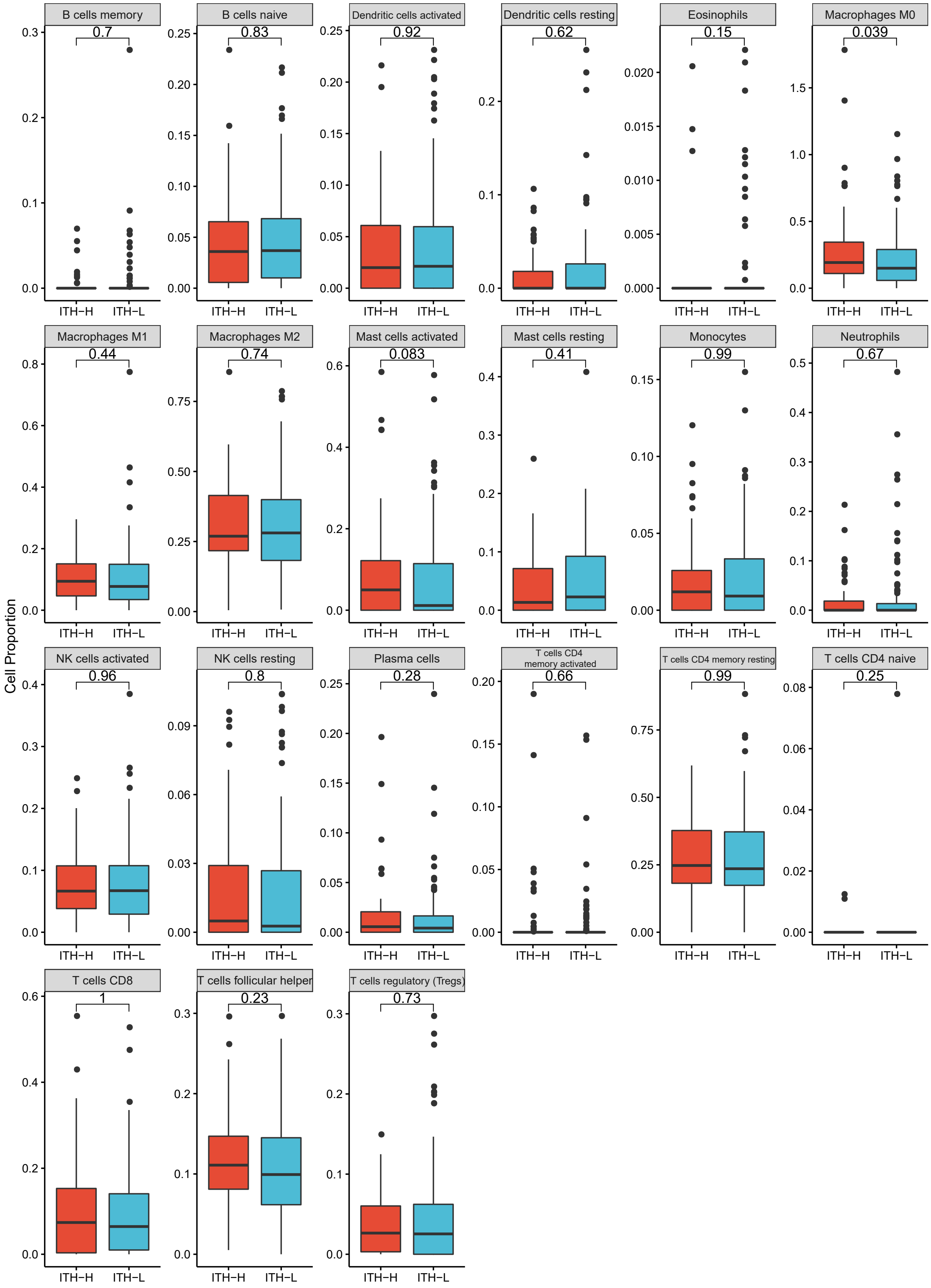

Cell Proportion
B cells memory
0.7
B cells naive
0.83
Dendritic cells activated
0.92
Dendritic cells resting
0.62
Eosinophils
0.15
Macrophages M0
0.039
Macrophages M1
0.44
Macrophages M2
0.74
Mast cells activated
0.083
Mast cells resting
0.41
Monocytes
0.99
Neutrophils
0.67
NK cells activated
0.96
NK cells resting
0.8
Plasma cells
0.28
T cells CD4 memory activated
0.66
T cells CD4 memory resting
0.99
T cells CD4 naive
0.25
T cells CD8
1
T cells follicular helper
0.23
T cells regulatory (Tregs)
0.73
ITH−H
ITH−L

Fig. S15. Difference of infiltration of each lymphocyte type in TCGA-LUSC. Difference of infiltration of each lymphocyte type between ITH-H group and ITH-L group in TCGA-LUSC.

Fig. S16.

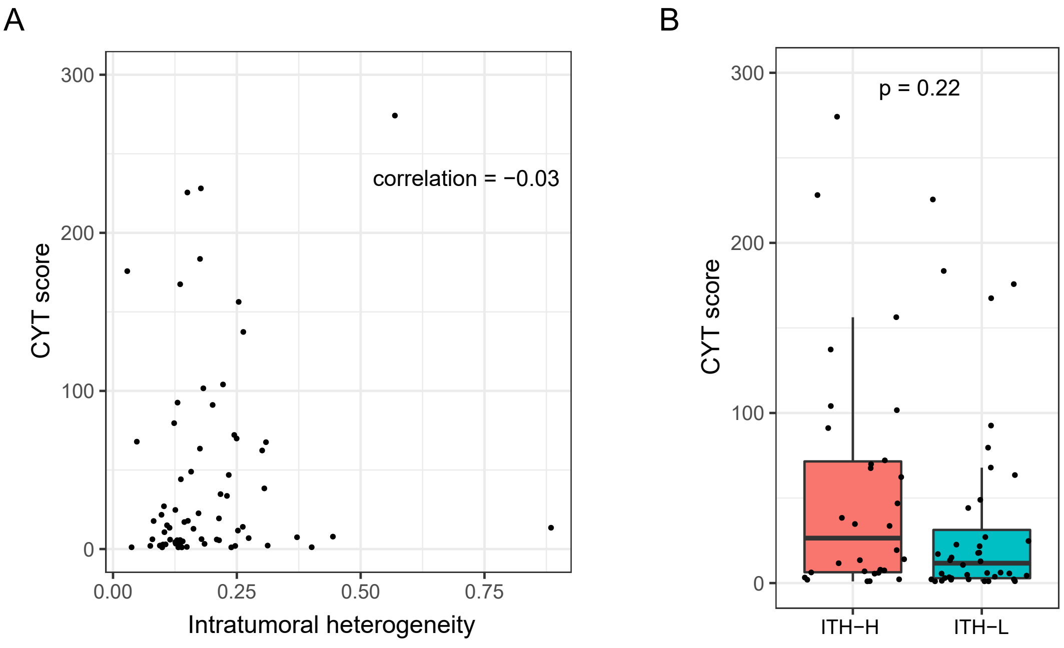

Fig. S16. Relationship between Cytolytic Activity score and ITH. a, Correlation between Cytolytic Activity (CYT) score and ITH value in Liu cohort. **b**, Distribution of CYT score of each patient between ITH-H group and ITH-L group in Liu cohort.
